# Crowd-sourced benchmarking of single-sample tumor subclonal reconstruction

**DOI:** 10.1038/s41587-024-02250-y

**Published:** 2024-06-11

**Authors:** Adriana Salcedo, Maxime Tarabichi, Alex Buchanan, Shadrielle M. G. Espiritu, Hongjiu Zhang, Kaiyi Zhu, Tai-Hsien Ou Yang, Ignaty Leshchiner, Dimitris Anastassiou, Yuanfang Guan, Gun Ho Jang, Mohammed F. E. Mootor, Kerstin Haase, Amit G. Deshwar, William Zou, Imaad Umar, Stefan Dentro, Jeff A. Wintersinger, Kami Chiotti, Jonas Demeulemeester, Clemency Jolly, Lesia Sycza, Minjeong Ko, PCAWG Evolution and Heterogeneity Working Group, Kerstin Haase, Jonas Demeulemeester, Clemency Jolly, Stefan C. Dentro, Ignaty Leshchiner, Moritz Gerstung, Maxime Tarabichi, Jeff Wintersinger, Amit G. Deshwar, Kaixian Yu, Santiago Gonzalez, Yulia Rubanova, Geoff Macintyre, David J. Adams, Pavana Anur, Rameen Beroukhim, Paul C. Boutros, David D. Bowtell, Peter J. Campbell, Shaolong Cao, Elizabeth L. Christie, Marek Cmero, Yupeng Cun, Kevin J. Dawson, Nilgun Donmez, Ruben M. Drews, Roland Eils, Yu Fan, Matthew Fittall, Dale W. Garsed, Gad Getz, Gavin Ha, Marcin Imielinski, Lara Jerman, Yuan Ji, Kortine Kleinheinz, Juhee Lee, Henry Lee-Six, Dimitri G. Livitz, Salem Malikic, Florian Markowetz, Inigo Martincorena, Thomas J. Mitchell, Ville Mustonen, Layla Oesper, Martin Peifer, Myron Peto, Benjamin J. Raphael, Daniel Rosebrock, S. Cenk Sahinalp, Adriana Salcedo, Matthias Schlesner, Steven Schumacher, Subhajit Sengupta, Ruian Shi, Seung Jun Shin, Lincoln D. Stein, Oliver Spiro, Ignacio Vázquez-García, Shankar Vembu, David A. Wheeler, Tsun-Po Yang, Xiaotong Yao, Ke Yuan, Hongtu Zhu, Wenyi Wang, Quaid D. Morris, Paul T. Spellman, David C. Wedge, Peter Van Loo, SMC-Het Participants, Alokkumar Jha, Tanxiao Huang, Tsun-Po Yang, Martin Peifer, S. Cenk Sahinalp, Salem Malikic, Ignacio Vázquez-García, Ville Mustonen, Hsih-Te Yang, Ken-Ray Lee, Yuan Ji, Subhajit Sengupta, Rudewicz Justine, Nikolski Macha, Schaeverbeke Quentin, Ke Yuan, Florian Markowetz, Geoff Macintyre, Marek Cmero, Belal Chaudhary, Ignaty Leshchiner, Dimitri Livitz, Gad Getz, Phillipe Loher, Kaixian Yu, Wenyi Wang, Hongtu Zhu, David C. Wedge, Quaid D. Morris, Kyle Ellrott, Peter Van Loo, Paul C. Boutros

**Affiliations:** 1 Department of Human Genetics, University of California, Los Angeles, CA USA; 2 Jonsson Comprehensive Cancer Center, University of California, Los Angeles, CA USA; 3 Institute for Precision Health, University of California, Los Angeles, CA USA; 4 Department of Medical Biophysics, University of Toronto, Toronto, Ontario Canada; 5 Ontario Institute for Cancer Research, Toronto, Ontario Canada; 6 The Francis Crick Institute, London, UK; 7 Wellcome Sanger Institute, Hinxton, UK; 8 Institute for Interdisciplinary Research, Université Libre de Bruxelles, Brussels, Belgium; 9 Oregon Health and Sciences University, Portland, OR USA; 10 Department of Computational Medicine and Bioinformatics, University of Michigan, Ann Arbor, MI USA; 11 Department of Systems Biology, Columbia University, New York, NY USA; 12 Center for Cancer Systems Therapeutics, Columbia University, New York, NY USA; 13 Department of Electrical Engineering, Columbia University, New York, NY USA; 14 Broad Institute of MIT and Harvard, Cambridge, MA USA; 15 Herbert Irving Comprehensive Cancer Center, Columbia University, New York, NY USA; 16 Department of Internal Medicine, University of Michigan, Ann Arbor, MI USA; 17 Department of Electronic Engineering and Computer Science, University of Michigan, Ann Arbor, MI USA; 18 Department of Computer Science, University of Toronto, Toronto, Ontario Canada; 19 VIB Center for Cancer Biology, Leuven, Belgium; 20 Department of Oncology, KU Leuven, Leuven, Belgium; 21 Big Data Institute, University of Oxford, Oxford, UK; 22 Manchester Cancer Research Center, University of Manchester, Manchester, UK; 23 Vector Institute, Toronto, Ontario Canada; 24 Computational and Systems Biology Program, Memorial Sloan Kettering Cancer Center, New York, NY USA; 25 Department of Genetics, The University of Texas MD Anderson Cancer Center, Houston, TX USA; 26 Department of Genomic Medicine, The University of Texas MD Anderson Cancer Center, Houston, TX USA; 27 Department of Pharmacology and Toxicology, University of Toronto, Toronto, Ontario Canada; 28 Department of Urology, University of California, Los Angeles, CA USA; 29 Broad Stem Cell Research Center, University of California, Los Angeles, CA USA; 30 California NanoSystems Institute, University of California, Los Angeles, CA USA; 31 Wellcome Trust Sanger Institute, Cambridge, UK; 32 European Molecular Biology Laboratory, European Bioinformatics Institute, Cambridge, UK; 33 University of Toronto, Toronto, Ontario Canada; 34 The University of Texas MD Anderson Cancer Center, Houston, TX USA; 35 Cancer Research UK Cambridge Institute, University of Cambridge, Cambridge, UK; 36 Molecular and Medical Genetics, Oregon Health and Science University, Portland, OR USA; 37 Dana-Farber Cancer Institute, Boston, MA USA; 38 University of California Los Angeles, Los Angeles, CA USA; 39 Peter MacCallum Cancer Centre, Melbourne, Victoria Australia; 40 University of Melbourne, Melbourne, Victoria Australia; 41 Walter and Eliza Hall Institute, Melbourne, Victoria Australia; 42 Department of Translational Genomics, Center for Integrated Oncology Cologne-Bonn, Medical Faculty, University of Cologne, Cologne, Germany; 43 Simon Fraser University, Burnaby, British Columbia Canada; 44 Vancouver Prostate Centre, Vancouver, British Columbia Canada; 45 German Cancer Research Center (DKFZ), Heidelberg, Germany; 46 Heidelberg University, Heidelberg, Germany; 47 Massachusetts General Hospital Center for Cancer Research, Charlestown, MA USA; 48 Department of Pathology, Massachusetts General Hospital, Boston, MA USA; 49 Harvard Medical School, Boston, MA USA; 50 Weill Cornell Medicine, New York, NY USA; 51 New York Genome Center, New York, NY USA; 52 University of Ljubljana, Ljubljana, Slovenia; 53 NorthShore University HealthSystem, Evanston, IL USA; 54 The University of Chicago, Chicago, IL USA; 55 University of California Santa Cruz, Santa Cruz, CA USA; 56 University of Cambridge, Cambridge, UK; 57 Organismal and Evolutionary Biology Research Programme, Department of Computer Science, Institute of Biotechnology, University of Helsinki, Helsinki, Finland; 58 Carleton College, Northfield, MN USA; 59 Princeton University, Princeton, NJ USA; 60 Indiana University, Bloomington, IN USA; 61 Korea University, Seoul, Republic of Korea; 62 Computational Oncology, Memorial Sloan Kettering Cancer Center, New York, NY USA; 63 Irving Institute for Cancer Dynamics, Columbia University, New York, NY USA; 64 Human Genome Sequencing Center, Baylor College of Medicine, Houston, TX USA; 65 School of Computing Science, University of Glasgow, Glasgow, UK; 66 Oxford NIHR Biomedical Research Centre, Oxford, UK; 67 Manchester Cancer Research Centre, University of Manchester, Manchester, UK; 68 Insight Centre for Data Analytics, NUIG, Galway, Ireland; 69 Bioinfo, HaploX Biotechnology, Shenzhen, China; 70 University of Cologne, Cologne, Germany; 71 Simon Fraser University, Vancouver, British Columbia Canada; 72 Memorial Sloan Kettering Cancer Center, New York, NY USA; 73 Columbia University, New York, NY USA; 74 Wellcome Sanger Institute, Cambridge, UK; 75 University of Helsinki, Helsinki, Finland; 76 Levine Cancer Institute, Atrium Health, Charlotte, NC USA; 77 Department of of Medical Imaging and Intervention, Chang Gung Memorial Hospital at Linkou, Taoyuan City, Taiwan; 78 NorthShore University HealthSystem, Chicago, IL USA; 79 Bordeaux University, Bordeaux, France; 80 Centre National de la Recherche Scientifique (CNRS), Paris, France; 81 CRUK Cambridge Institute, University of Cambridge, Cambridge, UK; 82 Computational Medicine Center, Thomas Jefferson University, Philadelphia, PA USA; 83 University of North Carolina at Chapel Hill, Chapel Hill, NC USA

**Keywords:** Computational models, Cancer genomics, Genome informatics

## Abstract

Subclonal reconstruction algorithms use bulk DNA sequencing data to quantify parameters of tumor evolution, allowing an assessment of how cancers initiate, progress and respond to selective pressures. We launched the ICGC–TCGA (International Cancer Genome Consortium–The Cancer Genome Atlas) DREAM Somatic Mutation Calling Tumor Heterogeneity and Evolution Challenge to benchmark existing subclonal reconstruction algorithms. This 7-year community effort used cloud computing to benchmark 31 subclonal reconstruction algorithms on 51 simulated tumors. Algorithms were scored on seven independent tasks, leading to 12,061 total runs. Algorithm choice influenced performance substantially more than tumor features but purity-adjusted read depth, copy-number state and read mappability were associated with the performance of most algorithms on most tasks. No single algorithm was a top performer for all seven tasks and existing ensemble strategies were unable to outperform the best individual methods, highlighting a key research need. All containerized methods, evaluation code and datasets are available to support further assessment of the determinants of subclonal reconstruction accuracy and development of improved methods to understand tumor evolution.

## Abstract (web summary)

Benchmarking of tumor subclonal reconstruction algorithms finds that interalgorithm variability overwhelms intertumor variability and identifies key quality metrics.

## Main

Tumors evolve from normal cells through the sequential acquisition of somatic mutations. These mutations occur probabilistically, influenced by the cell’s chromatin structure and both endogenous and exogenous mutagenic pressures^1^. If specific mutations provide a selective advantage to a cell, then its descendants can expand within their local niche. This process can repeat over years or decades until a population of cells descended from a common ancestor (a clone) emerges showing multiple hallmarks of cancer^2,3^. Throughout this time, different tumor cell subpopulations (subclones) can emerge through drift or selective pressures across the population^4^. While the precise definition of clones and subclones can be context dependent, a useful and commonly used way to identify clones and subclones is through a common set of mutations shared by cells with a common ancestor^4^.

The evolutionary features of tumors are increasingly recognized to have clinical implications. Genetic heterogeneity has been associated with worse outcomes, larger numbers of mutations and therapy resistance^5–8^. The evolutionary timing of individual driver mutations influences the fraction of cancer cells that will be affected by therapies targeting them. The specific pattern of mutations and their timing can shed light on tumor etiology and sometimes predict therapeutic sensitivity^9^.

The process of inferring the quantitative features of an individual tumor’s (sub)clonal composition on the basis of the mutational features of its genome is called subclonal reconstruction^10^ and is a common approach to quantify aspects of tumor evolution. Numerous algorithms based on the allelic frequencies of somatic single-nucleotide variants (SNVs) and copy-number aberrations (CNAs) have been developed for this task. Many apply Bayesian inference^11–14^ but a broad variety of strategies have been developed^15–17^.

Subclonal reconstruction results can vary substantially from algorithm to algorithm^18^. Little is known about how tumor characteristics and technical parameters, such as depth of sequencing or accuracies of variant and copy-number calls, quantitatively influence the performance of subclonal reconstruction algorithms. It is even unclear how best to quantify algorithm accuracy^19^. There is a clear need to identify which subclonal reconstruction algorithms most accurately infer specific evolutionary features and what aspects of both the cancer itself and the DNA sequencing experiment most influence accuracy.

To address these questions, we applied a validated framework for simulating and scoring evolutionarily realistic cancers^19^ in a crowd-sourced benchmarking challenge to quantify the accuracy of 31 strategies for subclonal reconstruction against 51 extensively annotated tumor phylogenies. Using this library of interchangeable methods, we quantified algorithm performance and showed that only a small number of specific tumor features strongly influence reconstruction accuracy. These results and resources will improve the application of existing subclonal reconstruction methods and support algorithm enhancement and development.

## Results

### Challenge design

**Fig. 1:**
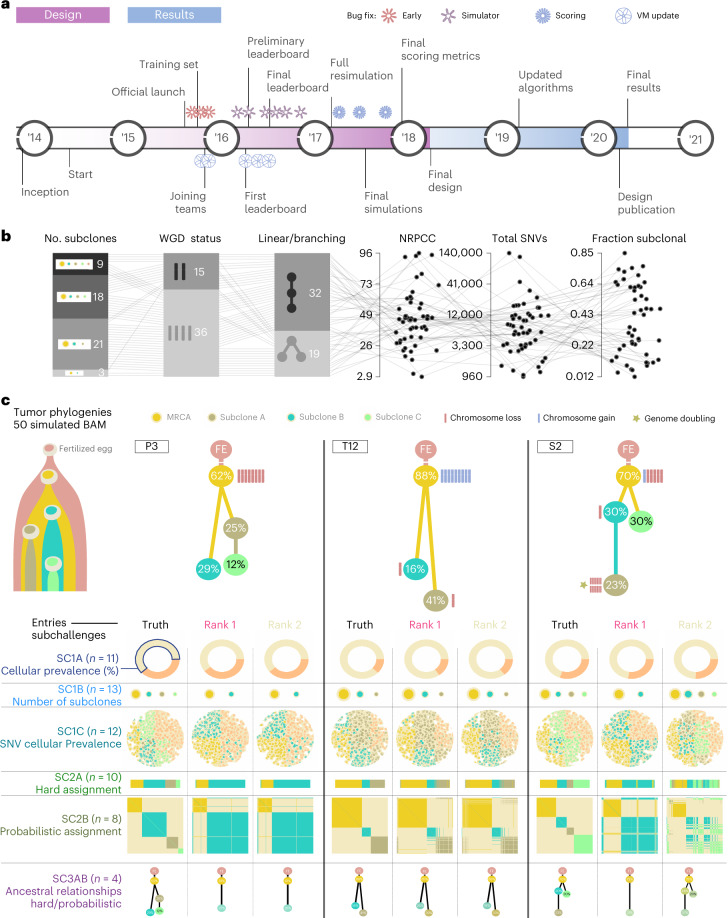
Design of the challenge. **a**, Timeline of the SMC-Het DREAM Challenge. The design phase started in 2014 with final reporting in 2021. VM, virtual machine. **b**, Simulation parameter distributions across the 51 tumors. From left to right: number of subclones, whole-genome doubling status, linear versus branching topologies, NRPCC, total number of SNVs and fraction of subclonal SNVs. **c**, Examples of tree topologies for three simulated tumors (P3, T12 and S2). For each simulated tumor, its tree topology is shown on top of the truth (column 1) and two example methods predictions (columns 2 and 3) for each subchallenge (rows). MRCA, most recent common ancestor.

**Extended Data Fig. 1:**
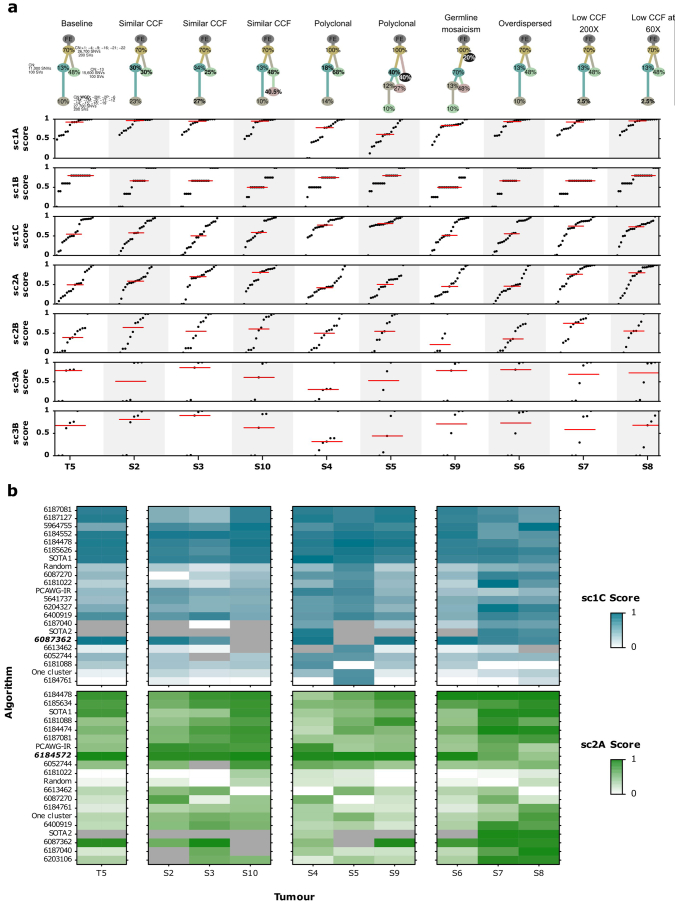
Design and scoring of special case tumours. **a**) Designs of special case tumours (top row) and their scores across SubChallenges. Each point in the strip plots represents an entry score and the red line shows the median (N=1160 {tumour, algorithm, SubChallenge} scores. **b**) Heatmap of scores for sc1C and sc2A for each entry on the corner case tumours. Tumour T5 is considered as the baseline. Top performing methods are shown in bold, italic text.

To benchmark methods for tumor subclonal reconstruction, we built upon the ICGC–TCGA (International Cancer Genome Consortium–The Cancer Genome Atlas) DREAM Somatic Mutation Calling Challenge and its tumor simulation framework (Fig. 1a)^19–21^. We designed 51 tumor phylogenies (Supplementary Fig. 1) to cover a wide range of biological and technical parameters (Fig. 1b). In total, 25 of these phylogenies were based on manually curated tumors from the Pan-Cancer Analysis of Whole Genomes (PCAWG) study^22^, while 16 were based on non-PCAWG tumors^13,23–28^ (the Somatic Mutation Calling Tumor Heterogeneity and Evolution Challenge (SMC-Het) cohort). The remaining ten were designed as variations of a single breast tumor, each testing a specific edge case or assumption of subclonal reconstruction algorithms (the special cases; Extended Data Fig. 1a)^13^. We supplemented these with a five-tumor titration series at 8×, 16×, 32×, 64× and 128× coverage^19^ (the titration series). For each tumor design, we simulated normal and tumor BAM files using BAMSurgeon^19^ and then used the Genome Analysis Toolkit (GATK) MuTect^29^ to identify somatic SNVs and Battenberg^13^ to identify somatic CNAs and estimate tumor purity. These were provided as inputs to participating groups, who were blinded to all other details of the tumor genome and evolutionary history.

**Extended Data Fig. 2:**
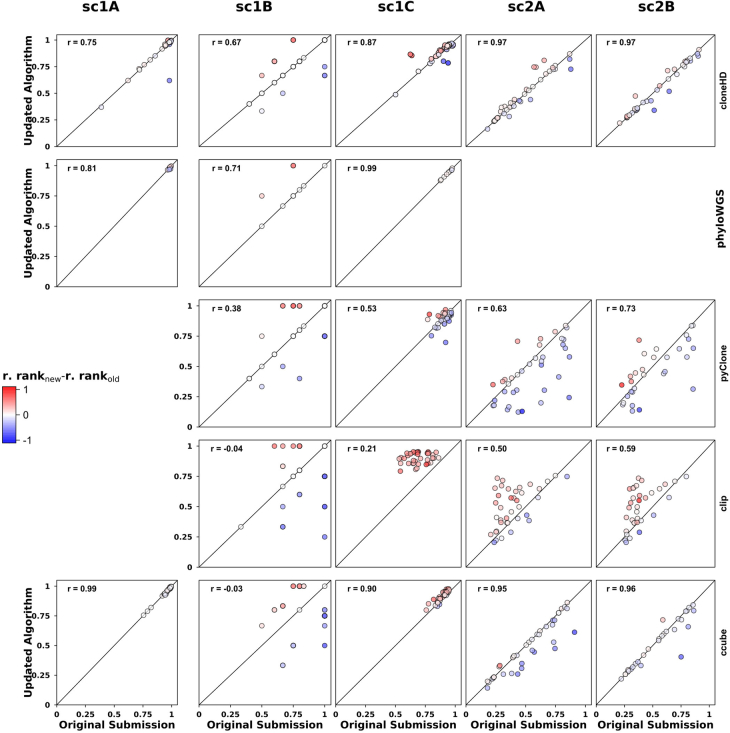
Effects of algorithm version updates. Updated (y-axis) and original (x-axis) for five algorithms on the SMC-Het tumours. Point colour reflects the difference in the algorithm’s relative rank (r. rank) for that tumour.

Participating teams submitted 31 containerized workflows that were executed in a reproducible cloud architecture^30^. Organizers added five reference algorithms: an assessment of random chance predictions, the PCAWG ‘informed brute-force’ clustering^31^, an algorithm that placed all SNVs in a single cluster at the variant allele frequency (VAF) mode and two state-of-the-art (SOTA) algorithms (DPClust^13^ and PhyloWGS^11^). Each method was evaluated on seven subchallenges evaluating different aspects of subclonal reconstruction: sc1A, purity; sc1B, subclone number; sc1C, SNV cellular prevalences (CPs); sc2, clusters of mutations; sc3, phylogenies (Fig. 1c). Note that both subchallenges 2 and 3 have paired deterministic (‘hard’) (sc2A and sc3A) and probabilistic (‘soft’) (sc2B and sc3B) tasks. A Docker container for each entry is publicly available from Synapse (https://www.synapse.org/#!Synapse:syn2813581/files/). Each prediction was scored using an established framework, with scores normalized across methods within {tumor, subchallenge} tuples to range from zero to one^19^. Runs that generated errors and produced no outputs, that produced malformed outputs or that did not complete within 21 days on a compute node with at least 24 central processing units (CPUs) and 200 GB of random-access memory (RAM) were deemed failures (2,189 runs; Supplementary Table 1). Failures mainly occurred for two tumors with over 100,000 SNVs. To ensure that our conclusions were consistent across software versions, we executed updated versions for five algorithms (Extended Data Fig. 2 and Supplementary Table 1). Differences were modest (*r* = 0.74) but varied across subchallenges and algorithms; updates particularly influenced assessments of subclone number (sc1B; *r* = 0.34). In total, we considered 11,432 runs across the seven subchallenges (Supplementary Table 1) and refined these to 6,758 scores after eliminating failed runs and highly correlated submissions (*r* > 0.75) from the same team, while considering only submissions made during the initial challenge period (Methods and Supplementary Tables 2 and 3).

### Top-performing subclonal reconstruction methods

**Fig. 2:**
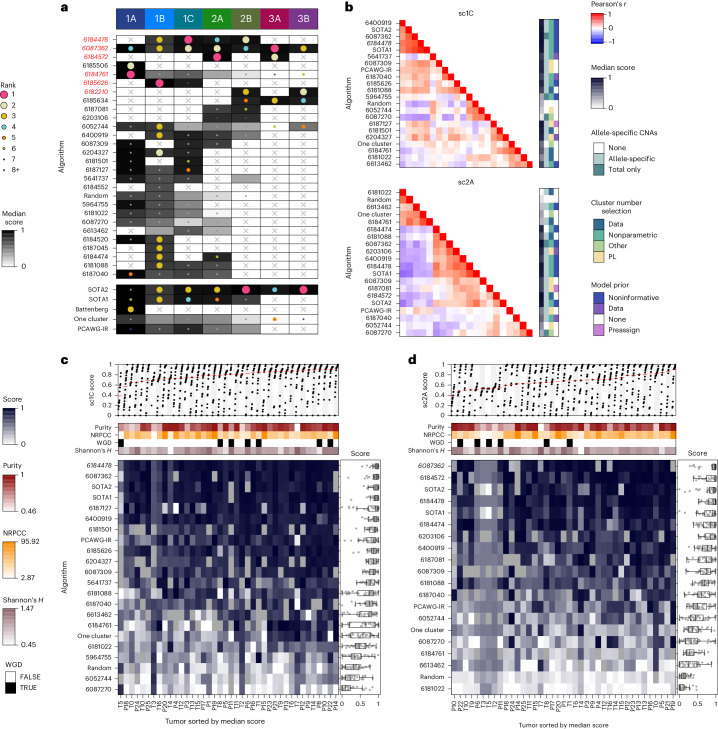
Overview of algorithm performance. **a**, Ranking of algorithms on each subchallenge based on median score. The size and color of each dot shows the algorithm rank on a given subchallenge, while the background color reflects its median score. The winning submissions are highlighted in red, italic text. **b**, Algorithm score correlations on sc1C and sc2A with select algorithm features. The top-performing algorithm for each subchallenge is shown in italic text. **c**,**d**, Algorithm scores on each tumor for sc1C (*n* = 805) {tumor, algorithm} (**c**) and sc2A (*n* = 731 {tumor, algorithm} (**d**) scores. Bottom panels show the algorithm scores for each tumor with select tumor covariates shown above The distribution of relative ranks for each algorithm across tumors is shown in the left panel. Boxes extend from the 0.25 to the 0.75 quartile of the data range, with a line showing the median. Whiskers extend to the furthest data point within 1.5 times the interquartile range. Top panels show scores for each tumor across algorithms, with the median highlighted in red. Tumors are sorted by difficulty from highest (left) to lowest (right), estimated as the median score across all algorithms.

**Extended Data Fig. 3:**
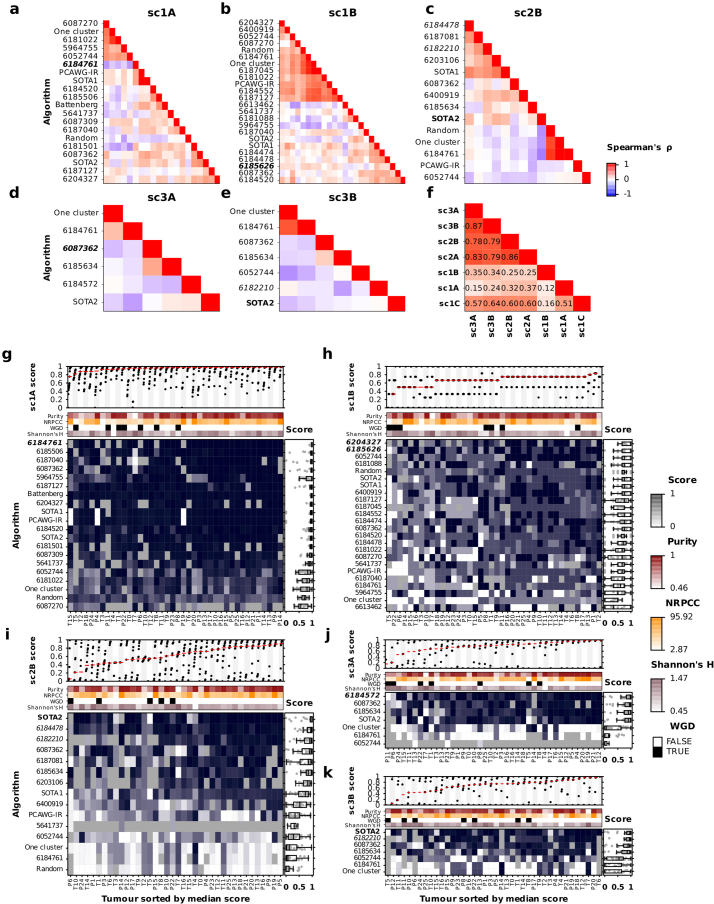
Overview of SubChallenge scores. **a-e**) Correlation in scores among algorithms. Each row and column is an entry for a specific SubChallenge, with colour reflecting Spearman’s ρ between entries across the main 40 SMC-Het tumours (excluding the corner cases and two tumours with > 100k SNVs where only five algorithms generated outputs), or the subset both algorithms successfully executed upon. Algorithms are clustered by correlation. Columns are sorted left-to-right in the same order that rows are top-to-bottom, thus values along the principal diagonal are all one. Top performing algorithms are shown in bold, italic text. **f**) Correlation in scores among SubChallenges **g-k**) Scores for each tumour for SubChallenge 1A including Battenberg purity estimates as a reference (N=719 {tumour, algorithm} scores. **g**) sc1B (N=895 {tumour, algorithm} scores. **h**) sc2B (N=471 {tumour, algorithm} scores. **i**) sc3A (N=218 {tumour, algorithm} scores. **j**) and sc3B (N=234 {tumour, algorithm} scores. **k**) on the SMC-Het tumours. The top performing algorithm for each SubChallenge is shown in bold text and the winning submission is shown in italic. Bottom panels show algorithm scores for each tumour with select tumour covariates shown above. The distribution of relative ranks for each algorithm across tumours is shown in the left panel. Boxes extend from the 0.25 to the 0.75 quartile of the data range with a line showing the median. Whiskers extend to the furthest data point within 1.5 times the interquartile range. Top panels show scores for each tumour across algorithms with the median highlighted in red.

**Extended Data Fig. 4:**
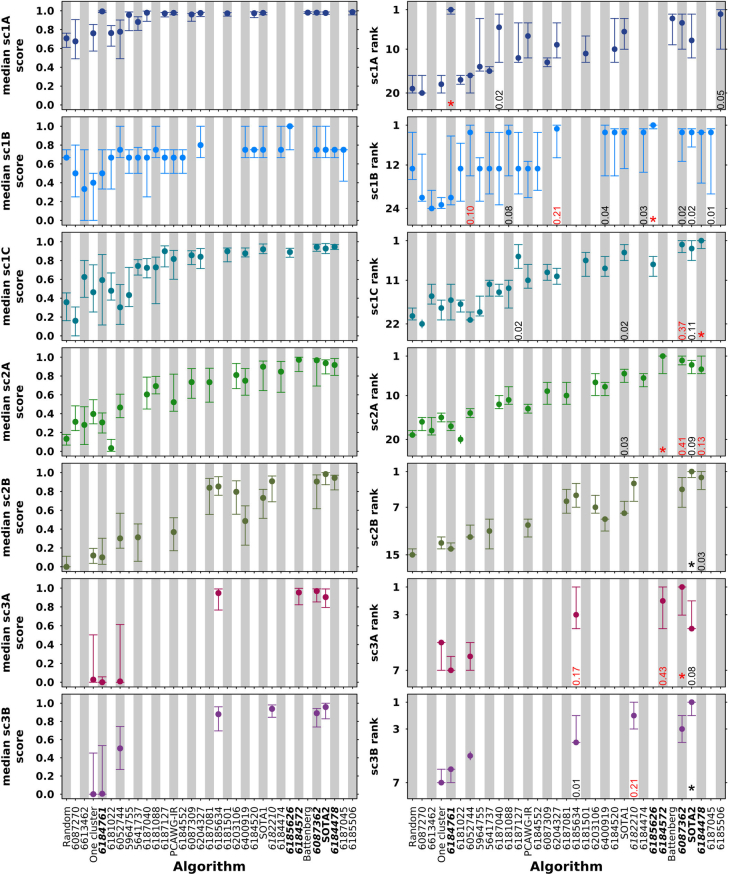
Rank generalizability assessment. To evaluate generalizability of ranks and differences amongst algorithms, bootstrap 95% confidence intervals were generated for median scores (left column) and ranks (right column) based on 1000 resamples. The observed median and rank and error bars representing 95% bootstrap confidence intervals are shown. The top ranking algorithms are marked with a star for each SubChallenge and highlighted in bold on the x-axis. Winning submissions are highlighted in red. For any entry with confidence intervals overlapping those of the top ranking algorithm, one-sided bootstrap P-values comparing the rank of that algorithm to the top ranking algorithm are shown: P(rankentry ≤ rankbest). P-values for equivalent top performers (P>0.1) are highlighted in red. Algorithms are sorted by the median of their relative rank (rank/maximum rank) on each SubChallenges and top performing algorithms are highlighted in bold. Battenberg is included as a reference for sc1A.

We ranked algorithms on the basis of median scores across all tumors; no single eligible entry was the top performer across multiple subchallenges (Fig. 2a). For each subchallenge, a group of algorithms showed strong and well-correlated performance (Fig. 2b and Extended Data Fig. 3a–e), suggesting multiple near-equivalent top performers. Therefore, we bootstrapped across tumors to test the statistical significance of differences in ranks (that is, to assess rank_entry_ < rank_best_ and assign a *P* value under the null hypothesis that rank_entry_ = rank_best_). sc1A and sc2B had single top-performing submissions, while two statistically indistinguishable (*P* > 0.1) submissions were identified for sc1B and sc1C, along with three for sc2A (Extended Data Fig. 4 and Table 1). The top performer for sc1A used copy-number calls alone to infer purity, while the second-best and statistically indistinguishable (P16) sc1A method used a consensus of purity estimates from both copy-number and SNV clustering.

**Table 1:** Top-performing methods for each subchallenge (subchallenges where the method was a top performer are indicated with X)

Algorithm	Associated IDs	sc1A	sc1B	sc1C	sc2A	sc2B	sc3A	sc3B	Reference
Object integration	6184761	X							Not available
PhylogicNDT	6184478			X	X	X			^31^
GISL	6185626, 6087362	X	X	X	X		X		Supplementary Note 1
CCube	6204327		X						^44^
FastClone	6184572, 6182210				X		X	X	^45^

Seven algorithms were submitted to the phylogenetic reconstruction tasks (sc3A and sc3B). Multiple algorithms were statistically indistinguishable as top performers in both challenges (Extended Data Fig. 4) but accuracy differed widely across and within tumors. Two examples of divergent predictions are given in Supplementary Fig. 2a,b. The predicted and true phylogenies for all tumors can be found at https://mtarabichi.shinyapps.io/smchet_results/; true phylogenies are provided in Supplementary Fig. 1. Algorithms differed in their ability to identify branching phylogenies (Supplementary Fig. 2c) and in their tendency to merge or split individual nodes (Supplementary Fig. 2d). Parent clone inference errors shared similarities across algorithms; the ancestor inference for SNVs within a node was more likely to be correct if the node was closely related to the normal (that is, if it was the clonal node or its child) (Supplementary Fig. 2e,f). When algorithms inferred the wrong parent for a given SNV, most assignment errors were to closely related nodes (Supplementary Fig. 2g). As expected, these results emphasize that single-sample phylogenetic reconstruction was most reliable for variants with higher expected alternate read counts (that is, clonal variants) and their direct descendants; detailed phylogenies varied widely across tumors and algorithms.

The scores of methods across subchallenges were correlated (Extended Data Fig. 3f). This was in part driven by patterns in the set of submissions that tackled each problem and in part by underlying biological relationships among the problems. For example, sc1C, sc2A and sc2B assessed different aspects of SNV clustering and their scores were strongly correlated with one another but not with tumor purity estimation scores (sc1A). Rather, numerous algorithms scored highly on sc1A, suggesting that different approaches were effective at estimating CP (Extended Data Fig. 4).

### Algorithm performance is largely invariant to tumor biology

To understand the determinants of the variability in algorithm performance between and within tumors, we considered the influence of tumor intrinsic features. We ranked tumors by difficulty, quantified as the median score across all algorithms for each subchallenge (Fig. 2c,d and Extended Data Fig. 3g–k). The most and least difficult tumors differed across subchallenges (Supplementary Fig. 3a) and tumor ranks across subchallenges were moderately correlated (Supplementary Fig. 3b). sc2A and sc2B were the most (*ρ* = 0.61) while sc1C and sc3B were the least correlated (*ρ* = −0.10).

**Extended Data Fig. 5:**
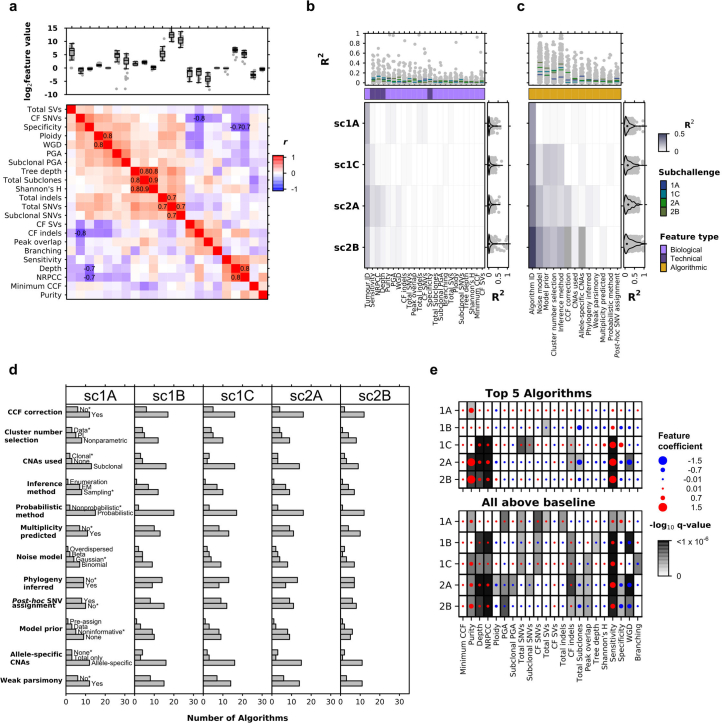
Tumour feature score associations. **a**) Correlations among tumour features and their distributions (boxplot, top). Boxes extend from the 0.25 to the 0.75 quartile of the data range with a line showing the median. Whiskers extend to the furthest data point within 1.5 times the interquartile range. N=42 tumours. NRPCC is number of reads per chromosome copy; CCF is cancer cell fraction; CF is clonal fraction (proportion of mutations in the clonal node); PGA is percent of the genome with a copy number aberration after correcting for ploidy. See Methods for detailed descriptions of each. **b,c**) Score variance explained by univariate generalized linear models (β-regressions with a logit link) for scores generated with tumour (**b**) and algorithm (**c**) features. Models were fit on scores from all algorithms ranking above the one cluster solution on a given SubChallenge. Heatmap shows R2 for univariate GLMs for features (x-axis) on SubChallenge score (y-axis) on the full dataset, gray indicates missing values where models could not be run. The right and upper panels show the marginal R2 distributions generated when running the univariate models separately on each algorithm and tumour (for tumour and algorithm features, respectively). Tumour and algorithm ID were not included in the marginal models as the number of levels would be equivalent to the number of observations in the data subset. Lines show the median R2 for each feature across the marginal models for each SubChallenge. **d**) Distribution of algorithm features. **e**) Results of generalized linear models for tumour features on scores (β regression with a logit link) that controlled for algorithm-ID. The size of the dots shows the effect size and the background colour shows the two-sided GLM Wald test P-value after FDR adjustment. Effect size interpretation is similar to that of a logistic regression, representing a one unit change in the log ratio of the score relative to its distance from a perfect score (that is βx=log(score/(1-score)). The bottom panel shows the results of modes fit on the full dataset. The top panel shows the same bi-variate models were fit on scores from the top five algorithms.

To determine whether specific aspects of tumor biology influence reconstruction accuracy, we identified 18 plausible tumor characteristics. We supplemented these with four features that represent key experimental or technical parameters (for example, read depth; Supplementary Table 2). These 22 ‘data-intrinsic’ features were generally poorly or moderately correlated to one another, with a few expected exceptions such as ploidy being well correlated with whole-genome duplication (WGD; Extended Data Fig. 5a). For each subchallenge, we assessed the univariate associations of each feature with the pool of scores from all algorithms that ranked above the one-cluster solution (Extended Data Fig. 5b). As a reference, we also considered the tumor identifier (ID), which captures all data-intrinsic features as a single categorical variable. We focused on the subchallenges with large numbers of submissions and where scores could be modeled as continuous proportions using β regression (Methods). Individual data-intrinsic features explained a small fraction of the variance for sc1A, sc1C, sc2A and sc2B. Tumor ID explained ~15% of the variance in scores and no individual feature explained over 10%, suggesting that data-intrinsic features were not exerting consistently large influences on subclonal reconstruction accuracy across algorithms.

**Fig. 3:**
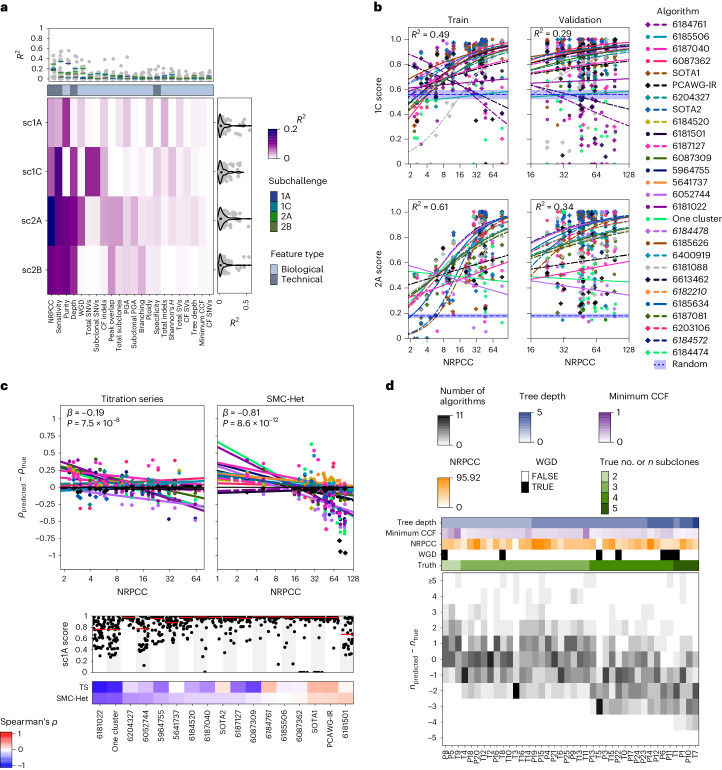
Tumor features influence subclonal reconstruction performance and biases. **a**, Score variance explained by univariate regressions for the top five algorithms in each subchallenge. The heatmap shows the *R*^2^ values for univariate regressions for features (*x* axis) on subchallenge score (*y* axis) when considering only the top five algorithms. The right and upper panels show the marginal *R*^2^ distributions generated when running the univariate models separately on each algorithm, grouped by subchallenge (right) and feature (upper). Lines show the median *R*^2^ for each feature across the marginal models for each subchallenge. **b**, Models for NRPCC on sc1C and sc2A scores when controlling for algorithm ID. The left column shows the model fit in the training set composed of titration-series tumors (sampled at five depths each) and five additional tumors (*n* = 10 individual tumors). The right column shows the fit in the test set (*n* = 30 tumors, comprising the remaining SMC-Het tumors after removing the edge cases). Blue dotted lines with a shaded region show the mean and 95% confidence interval based on scoring ten random algorithm outputs on the corresponding tumor set. The top-performing algorithm for each subchallenge is shown in italic text. **c**, Effect of NRPCC on purity error. The top panels show the purity error with NRPCC accounting for algorithm ID with fitted regression lines. The sc1A scores across tumors for each algorithm are shown in the panel below. The bottom heatmap shows Spearman’s *ρ* between purity error and NRPCC for each algorithm. The winning entry is shown in bold text. Two-sided *P* values from linear models testing the effect of NRPCC on sc1A error (with algorithm ID) are shown. TS, titration series. **d**, Error in subclone number estimation by tumor. The bottom panel shows the subclone number estimation error (*y* axis) for each tumor (*x* axis) with the number of algorithms that output a given error for a given tumor. Tumor features are shown above. See Methods for detailed descriptions of each of these.

We hypothesized that data-intrinsic features might, therefore, exhibit a method-specific effect that would be clearer in algorithms with generally strong performance. We repeated this univariate analysis on scores from the top five algorithms in each subchallenge, which were moderately correlated (Supplementary Fig. 3c). This modestly enhanced the strength of the detected associations. In sc1C, the varying sensitivity of SNV detection across tumors (relative to the simulated ground truth) explained 15.7% of variance in accuracy (Fig. 3a). In sc2A, the read depth adjusted for purity and ploidy (termed NRPCC, number of reads per chromosome copy^10^) explained 19.8% of the variance across tumors. The total number of SNVs and the number of subclonal SNVs explained 9.3% and 9.2% of the variance for sc1C, as might be expected, because both define the resolution for subclonal reconstruction^10^. These results indicate that data-intrinsic features either explained little of the variability in subclonal reconstruction accuracy or did so in ways that differed widely across algorithms.

### Algorithmic and experimental choices drive accuracy

Given the relatively modest impact of data-intrinsic features on performance, we next focused on algorithm-intrinsic features. We first modeled performance as a function of algorithm ID, which captures all algorithmic features. Algorithm choice alone explained 19–35% of the variance in scores in each subchallenge (Extended Data Fig. 5c). This exceeded the ~15% explained by tumor ID, despite our assessment of more tumors than algorithms.

To better understand the effect of algorithm choice, we quantified 12 algorithm characteristics. For example, we annotated whether each method adjusted allele frequencies for local copy number (Extended Data Fig. 5d). The variance explained by the most informative algorithm feature was 1.5–3 times higher than that of the most informative tumor feature (Extended Data Fig. 5c). Our analysis highlighted Gaussian noise models as particularly disadvantageous for SNV coclustering (sc2A) relative to binomial or β binomial noise models (generalized linear model (GLM) *B*_Gaussian_ = −0.98, *P* = 1.43 × 10^−15^, *R*^2^ = 0.11). This trend became stronger when we compared algorithms with Gaussian noise models to those with binomial noise models and adjusted for tumor ID (*B*_Gaussian_ = −1.11, *P* < 2 × 10^−16^, *R*^2^ = 0.35).

The strong impact of algorithm choice on performance led us to hypothesize that data-intrinsic features show algorithm-specific influences on performance. Therefore, we developed multivariate models to control for algorithm ID when modeling data-intrinsic features. After making this change, SNV caller sensitivity, tumor purity and experimental read depth were significantly associated with increased scores for nearly all subchallenges (*q* < 0.05). These associations were consistent whether we analyzed all algorithms that exceeded the baseline (Extended Data Fig. 5e) or only the top five algorithms for each subchallenge (Supplementary Fig. 3d). Our results show that algorithm choice was the strongest driver of subclonal reconstruction accuracy, followed by technical data-intrinsic features. Biological data-intrinsic features were weak determinants of subclonal reconstruction accuracy.

### Optimizing experimental design for subclonal reconstruction

Most data-intrinsic features reflect aspects of tumor biology not known a priori. In contrast, the main controllable technical feature is sequencing coverage. We investigated the sensitivity of subclonal reconstruction to this experimental design choice by considering NRPCC. By adjusting sequencing coverage for tumor purity and ploidy, NRPCC provides an excellent estimate of power in subclonal reconstruction^10^. We modeled the relationship between NRPCC and SNV coclustering subchallenge scores (sc1C and sc2A) using a GLM in which we controlled for algorithm ID, because of the strong influence of this feature in our univariate analyses above. We fit the model on five tumors with a coverage titration series (five points per tumor^19^) and on five randomly selected tumors, leading to 373 scores from these ten tumors. We then assessed model generalizability on 466 scores from 30 tumors. Nine edge cases and two tumors with a high mutation burden (>50,000 SNVs) were excluded from both the training and testing cohorts. As expected, higher NRPCC increased sc1C and sc2A scores for most algorithms (Fig. 3b). Increasing NRPCC improves coclustering by reducing read-sampling noise, thereby improving subclone resolution^10,31^. We observed an unexpected saturation effect; at high NRPCC, most variability in scores was because of differences among algorithms. These data quantify a clear benefit to tumor sequencing to an NRPCC of at least 32 for subclonal reconstruction from a single sample across the range of algorithms tested here.

**Extended Data Fig. 6:**
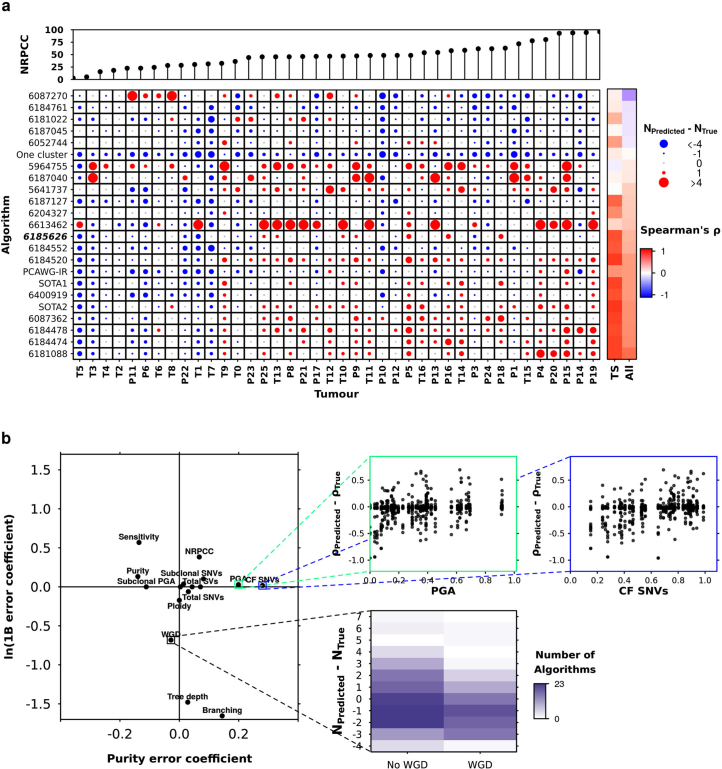
Mutational feature error associations. **a**) Error in subclone number estimation for each algorithm on each tumour (center). Top panel plot shows NRPCC for each tumour. Right panel shows subclone number estimation error correlations with NRPCC. The top performing algorithm for SubChallenge sc1B is shown in bold italic text. **b**) Coefficient from penalized regression models for tumour features on purity estimation error (x-axis) and subclone number estimation error (y-axis).

We replicated these analyses for estimation of tumor purity (sc1A). Lower NRPCC was associated with an overestimation of tumor purity (sc1A) in both the titration-series and the SMC-Het cohort (Fig. 3c). This likely occurred because, in low-coverage sequencing data, SNVs detected on a few reads were indistinguishable from background data. These false negatives led to a truncated binomial distribution and overestimation of the average frequencies of detected SNV clusters^10,31^. Conversely, high NRPCC increased the number of subclonal mutations detected, causing some algorithms to underestimate purity (especially the naive one-cluster and random algorithms). In a similar way, NRPCC influenced the prediction of subclone number (sc1B). More algorithms underpredicted the number of subclones as the tree depth and the true subclone number increased (Fig. 3d; *B*_tree depth_ = −1.18, *P* = 1.60 × 10^−41^, ordinal regression, likelihood ratio test), suggesting there was a limit to how many subclones could be distinguished at a given NRPCC. The number of subclones predicted increased with NRPCC for a given tumor for most algorithms (Extended Data Fig. 6a; *B* = 0.71, *P* = 2.99 ×10^−24^). These data emphasize that it is critical to report NRPCC and interpret estimates of tumor subclonal diversity in that context.

Lastly, we asked whether other tumor features might bias the prediction of purity and subclone number. We used multivariate penalized regression with leave-one-out cross-validation to model sc1A and sc1B errors. After controlling for algorithm ID, the sc1A model explained 40.1% of the variance and the sc1B model explained 57.1%. The multivariate model for purity estimation error highlighted that increasing SNV clonal fraction (CF) and percentage genome altered (PGA) reduced the purity underestimation errors but algorithms were more likely to overestimate purity when the true purity was low (Extended Data Fig. 6b). The subclone number error model showed that algorithms were more likely to underestimate the number of subclones if there was a WGD. These results suggest that increasing power (that is, NRPCC) is especially important if there is a priori knowledge that a given tumor or tumor type is prone to low purity, CF or PGA or is likely to harbor a WGD^10,31^. These results also confirmed NRPCC as a crucial study design parameter that should be considered when interpreting subclonal reconstruction results.

### Sources of error in SNV CP estimation

**Fig. 4:**
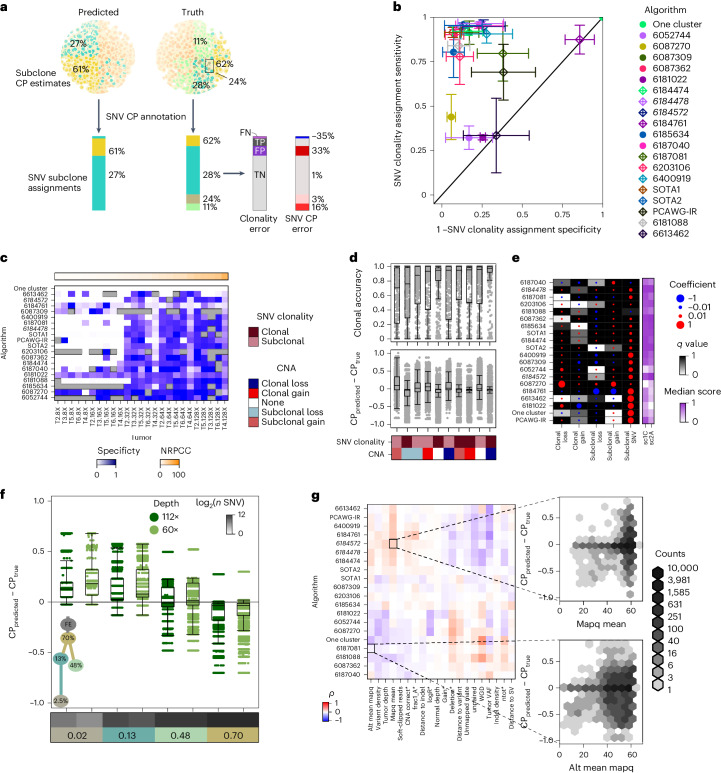
Impacts of genomic features on SNV subclonality predictions. **a**, Schematic showing how outputs from sc1C and sc2A were used to annotate SNV CP for each entry. FN, false negative; FP, false positive; TN, true negative; TP, true positive. **b**, Mean clonal SNV detection sensitivity and specificity for each algorithm with standard errors (*n* = 727 {tumor, algorithm} predictions). **c**, Clonal SNV detection *F* scores for each entry on each tumor. **d**, Top, clonal accuracy for each algorithm, CNA category and tumor tuple (*n* = 5,392); bottom, SNV CP estimation error for each algorithm (*n* = 4,868,460 {algorithm, SNV CP} predictions). Boxes extend from the 0.25 to the 0.75 quartile of the data range, with a line showing the median. Whiskers extend to the furthest data point within 1.5 times the interquartile range. **e**, Effect size and false discovery rate-adjusted two-sided *P* values from entry-specific linear regression models for SNV CP error by CNA type and SNV clonality with median sc1C and sc2A scores. Top performing entries are shown in italic text. **f**, SNV CP error grouped by subclone for a corner-case tumor simulated at two depths (*n* = 395,364 {algorithm, tumor, SNV} prediction errors). Boxes extend from the 0.25 to the 0.75 quartile of the data range, with a line showing the median. Whiskers extend to the furthest data point within 1.5 times the interquartile range. **g**, Correlation between BAM features and Battenberg output features with SNV CP error for each entry. Only features that had an absolute correlation > 0.1 are shown. Battenberg features are noted with a star and top-performing algorithms are highlighted in italic text.

Estimating the fraction of cancer cells in which each SNV occurs is one of the most fundamental goals of subclonal reconstruction, shedding light on the evolution of mutational processes in a tumor^3,31–33^. To understand errors in these estimates, we focused on the 20 algorithms that produced submissions for both sc1C and sc2A. For each tumor, we annotated the SNV subclone assignments (sc2A output) with the predicted CP for that subclone (sc1C output; Fig. 4a). Most algorithms accurately determined whether an SNV was clonal; 14 of 20 had both median specificity and sensitivity above 80% (Fig. 4b). Clonal assignment specificity increased with NRPCC, as more subclonal SNVs were correctly assigned, leading to improved accuracy (Fig. 4c and Supplementary Fig. 3a; *B*_log2(NRPCC)_ = 0.29, *q* = 3.11 × 10^−17^), and decreased with SNV caller precision (*B*_log2(precision)_ = −1.24 *q* = 1.94 × 10^−14^; Supplementary Fig. 4a). Accuracy also slightly decreased with mutational burden and tumor CF (Supplementary Fig. 4a).

The inference of SNV clonality was impacted by underlying copy-number states. Subclonal CNAs significantly reduced SNV clonality assignment accuracy relative to clonal CNAs after controlling for algorithm and tumor ID (*B*_subclonal CNA_ = −0.21, *P* = 1.14 × 10^−6^, GLM). SNVs that arose clonally in a region that then experienced a subclonal loss had the least accurate clonal estimates (Fig. 4d; *B*_clonal SNV ×__ subclonal loss_ = −0.33, *P* = 3.06 × 10^−2^; Supplementary Table 3). Subclonal losses on the mutation-bearing DNA copy reduced VAF, causing many algorithms to underestimate the CP of these SNVs (*W*_SNV clonal_ = 1.04 × 10^10^, *P* < 2.2 × 10^−16^, Wilcoxon rank-sum test for SNVs in subclonal deletions; Supplementary Table 3). Similarly, algorithms overestimated SNV CP in regions with subclonal gains and subclonal SNVs (*W*_SNV clonal_ = 2.96 × 10^9^, *P* < 2.2×10^−16^, Wilcoxon rank-sum test; Supplementary Table 4). This resulted in lower accuracy (*B*_subclonal SNV __× __subclonal gain_ = −0.32, *P* = 8.0 × 10^−3^, GLM; Fig. 4d and Supplementary Table 4). Biases in CP estimation because of CNAs differed among algorithms (Fig. 4e). To assess whether robustness to CNAs impacts performance, we associated the proportion of variance in SNV CP error explained by CNA status and SNV clonality in these models with algorithm score. Algorithms whose CP estimates were more robust to CNAs better estimated the overall subclonal CP distribution (sc1C; *ρ*_CNA_ = −0.43) and better coclustered SNVs (sc2A; *ρ*_CNA_ = −0.37; Supplementary Fig. 4b).

**Extended Data Fig. 7:**
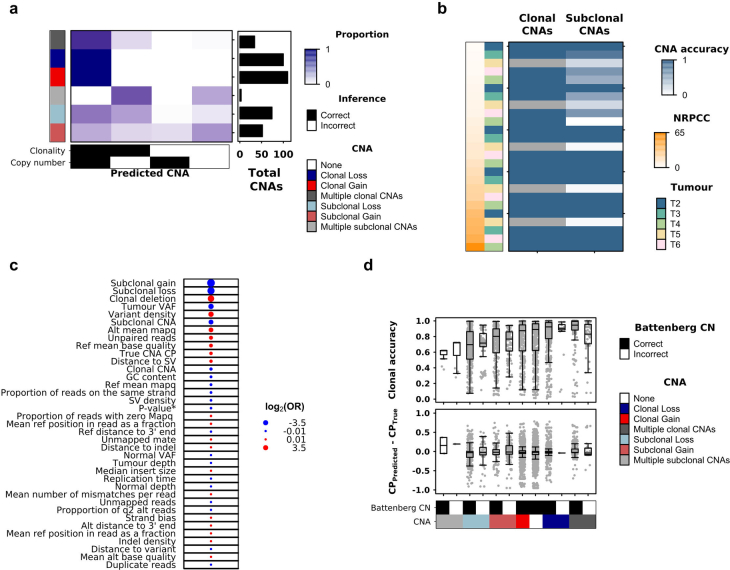
Battenberg CNA assessment. **a**) Battenberg errors for clonal and subclonal CNAs. The proportion of CNAs with correctly or incorrectly inferred clonality and copy number is shown in the heatmap. The total number of each type of CNA is indicated by the bar plot on the right. **b**) Battenberg accuracy in the titration series tumours. **c**) Effect sizes from a L1-regularized logistic regression for genomic features on Battenberg accuracy. **d**) Clonal accuracy for each entry and tumour combination (top) and SNV CP estimation error (bottom) for each entry shown as effect-sizes from an L1-regularlized logistic regression. Boxes extend from the 0.25 to the 0.75 quartile of the data range with a line showing the median. Whiskers extend to the furthest data point within 1.5 times the interquartile range.

Because subclonal CNAs can be difficult to detect, we investigated whether copy-number calling errors aggravated the effects of CNAs on estimation of CP. As expected, clonal CNA regions were nearly perfectly detected by our CNA caller (Battenberg; Extended Data Fig. 7a). By contrast, 7 of 68 subclonal losses and 25 of 48 subclonal gains were entirely missed and six more were misestimated. The accuracy of subclonal CNA detection was strongly influenced by tumor NRPCC (Extended Data Fig. 7b). Elastic net logistic regression showed that CNAs in low-CP subclones and SNP-poor regions were less accurately detected (Extended Data Fig. 7c). While Battenberg CNA calling errors did not significantly impact the accuracy of SNV clonality assignment, algorithms were more likely to overestimate CP for SNVs on segments with incorrect CNA states, with consistent direction of error biases (Extended Data Fig. 7d and Supplementary Table 5).

SNV features also shaped error profiles independently of CNAs. Almost all algorithms were more likely to overestimate the CP of subclonal SNVs (Fig. 4d,e) because of reduced power at lower tumor read depths^10,13,31^. Examining two edge-case tumors with identical architectures emphasized that this bias increased for lower subclone CP and NRPCC (Fig. 4f). To quantify how other sources of error in SNV and CNA calls propagate to subclonal reconstruction, we derived 53 measures of variant call quality from the BAM files, VCF files and Battenberg outputs (Methods) that we hypothesized could impact CP estimation and correlated them with CP error. Variant call quality was associated with CP error in patterns that varied across metrics and algorithms, with mean SNV mapping quality showing positive associations for many algorithms (Fig. 4g).

### Impact of neutral tail mutations on subclonal reconstruction

**Extended Data Fig. 8:**
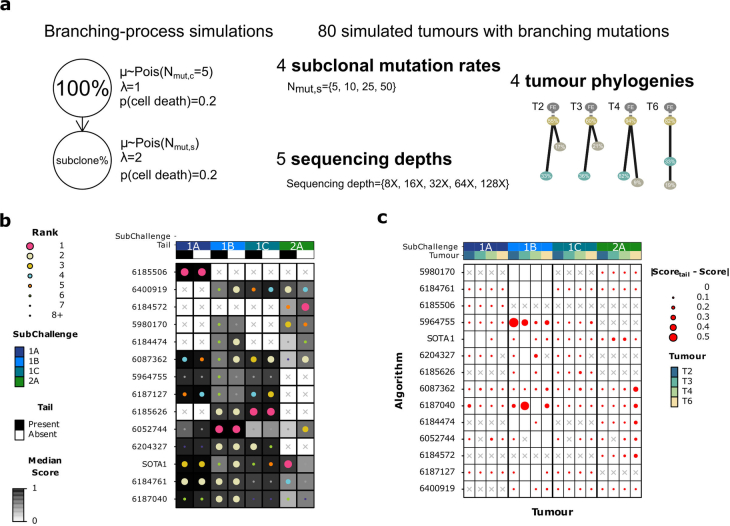
Effects of neutral tail simulation. **a**) Branching-process-based simulations adapted from Tarabichi et al. Nature Genetics 2018. The number of mutations at each cell division in the descendants of the most recent common ancestor is drawn from a Poisson distribution. We use a baseline of five mutations per cell division and vary the mutation rate in the subclones leading to neutral mutation tail size variation among subclones. We grow four tumours in silico with underlying phylogenies corresponding to T2, T3, T4, and T6 and track all neutral tail mutations. We simulate mutation calls in VCF format at increasing sequencing depths. **b**) Ranks of algorithms run on titration series tumours with and without the neutral tail at 25 neutral mutations per cell division. Ranks are based on the median normalized score across T2, T3, T4 and T6 and across depths (8x, 16x, 32x, 64x, 128x). **c**) Mean absolute difference in scores before and after the addition of tail mutations for each algorithm at 25 neutral mutations per cell division across tumours and depths.

Recent work showed that the ever-growing tail of point mutations at ever lower frequency may impact subclonal reconstruction^16^. These so-called ‘neutral tails’ can be explicitly modeled in subclonal reconstruction; however, because of their low CP, their practical importance at conventional whole-genome sequencing (WGS) coverages has been unclear^34^. To quantify their impact, we inserted neutral tail mutations into four titration-series tumors. We used agent-based cell division^34^ to derive the number and prevalence of neutral mutations, varying the tumor’s overall mutation rate (Extended Data Fig. 8, Methods and Supplementary Note 2). We tested the five best algorithms for sc1A, sc1B, sc1C and sc2A (18 methods; 1,440 reconstructions).

**Extended Data Fig. 9:**
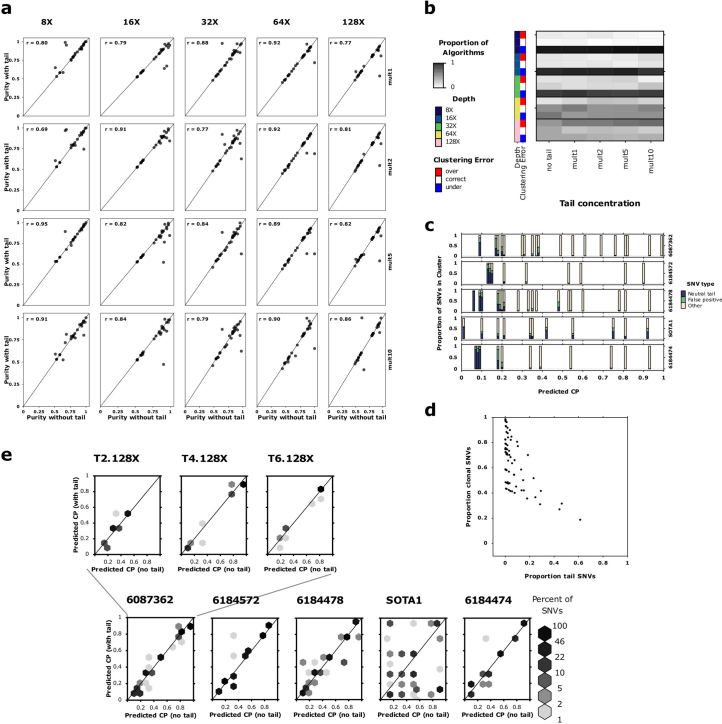
Error profiles of neutral tail simulation. **a**) Changes in purity estimates with the addition of neutral tails across algorithms with Pearson correlation shown. **b**) Subclone number estimation errors with increasing neutral tail mutation rates. Heatmap shows the proportion of algorithms that correctly, over- or under-estimate the number of subclones for each neutral tail mutation rate at each depth. **c**) Predicted subclone composition across the top five algorithms for sc2A at 128x and 25 neutral mutations per cell division. Each bar plot shows the CP of subclones predicted by a given algorithm across tumours and the proportion of SNVs in the subclone that are false positives, neutral tail mutations or neither. **d**) Proportion of SNVs predicted to be clonal by the top five algorithms for 2A against the true proportion of SNVs in the neutral tail across all neutral tail mutation rates and depths. **e**) Predicted CP of SNVs outside of the neutral tail for the top five ranking algorithms for sc2A at 128x and 25 neutral mutations per cell division. Each hexagon shows the proportion of SNVs within a tumour at given CP before and after adding the neutral tail mutations. Predicted CPs across all tumours for a given algorithm are aggregated within each plot (bottom row).

The effect of neutral tail mutations on subclonal reconstruction was generally modest in terms of both algorithm ranking and absolute scores (Extended Data Fig. 8), as well as error profiles (Extended Data Fig. 9). Their impact was observed at higher sequencing depths (>64×) where they tended to increase subclone number estimates (sc1B; *β* = 0.42, *P* = 3.52 ×10^−3^; Extended Data Fig. 9). At 128× coverage, most algorithms assigned tail mutations to low-VAF subclones with a high proportion of tail mutations and the predicted CP of SNVs outside the neutral tail was largely unaffected (Extended Data Fig. 9). At high depths, it may then be advantageous to explicitly account for tail mutations to avoid spurious low-VAF clusters.

Consistent with these findings, MOBSTER filtering, which identifies and removes tail mutations, significantly improved mutation assignment scores, especially as the branching tail size increased and at a depth > 64× (Supplementary Fig. 5). It reduced spurious clusters and removed many false-positive mutations. Thus, prefiltering could be incorporated into subclonal reconstruction pipelines when there is sufficient sequencing depth (>64×). The precise benefits of such filtering across a broad range of tumor and genomic contexts remain unclear but our results suggest that they may be worth defining, especially in the face of high-NRPCC sequencing.

### Pragmatic optimization of algorithm selection

**Fig. 5:**
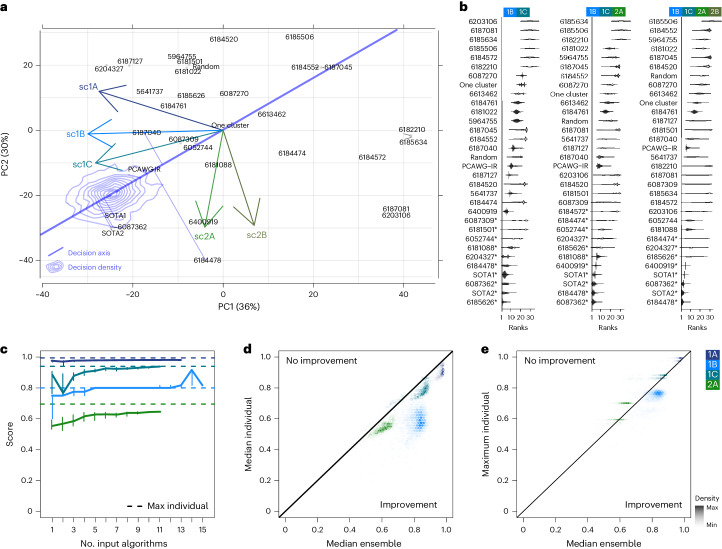
Performance across multiple algorithms and subchallenges. **a**, Projections of the algorithms and subchallenge axes in the principal components of the score space. A decision axis is also projected and corresponds to the axis of best scores across all subchallenges and tumors, when these are given equal weights. The five best methods according to this axis are projected onto it. A decision ‘brane’ in blue shows the density of decision axis coordinates after adding random fluctuations to the weights. **b**, Rank distribution of each method from 40,000 sets of independent random uniform weights given to each tumor and subchallenge in the overall score. From left to right: sc1B + sc1C; sc1B + sc1C + sc2A; sc1B + sc1C + sc2A + sc2B. Names of the algorithms have a star if they were ranked first at least once. **c**, Four subchallenges for each of which one ensemble approach could be used (sc1A, median; sc1B, floor of the median; sc1C, WeMe; sc2A, CICC; Methods); the median and the first and second tertiles (error bars) of the median scores are shown across tumors of independent ensembles based on different combinations of *n* methods (*n* is varied on the *x* axis). The dashed line represents the best individual score. **d**, Color-coded hexbin densities of median ensemble versus median individual scores across all combinations of input methods. The identity line is shown to delimit the area of improvement. **e**, Same as **d** for maximum individual scores instead of median scores.

We next sought to optimize algorithm selection across an arbitrary set of subchallenges. To visualize algorithm performance across all subchallenges, we projected both algorithms and subchallenges onto the first two principal components of the scoring space, explaining 66% of total variance (Fig. 5a). The blue ‘decision axis’ shows the axis of average score across subchallenges when all subchallenges were weighted equally and this axis was stable to small fluctuations in these weights (Fig. 5a). We randomly varied tumor and subchallenge weights 40,000 times across three groups of subchallenges: {sc1B, sc1C}, {sc1B, sc1C, sc2A} and {sc1B, sc1C, sc2A, sc2B} (Fig. 5b and Supplementary Note 3). Twelve algorithms (35%) reached a top rank within at least one study, while 22 (65%) were never ranked first. Because the choice of weights is ultimately user dependent, we created a dynamic web application for modeling the influence of different selections (https://mtarabichi.shinyapps.io/smchet_results/).

Ensemble approaches have previously been used in many different areas of biological data science to combine outputs from multiple algorithms and improve robustness^21,31,35,36^. They have not been widely explored for subclonal reconstruction, in part because many subclonal reconstruction outputs are complex and heterogeneous^31^. To assess whether ensemble approaches could improve subclonal reconstruction, we identified and ran ensemble methods for individual subchallenges based on median or voting approaches, which served as conservative baselines (Methods).

The median ensemble performance increased with the number of input algorithms for all subchallenges (Fig. 5c). Ensemble performance was more consistent across tumors for sc1A and sc1B when more input algorithms were used, as shown by the decreasing variance in scores (Supplementary Fig. 6). Ensemble approaches outperformed the best individual methods for sc1B but not for sc1A, sc1C or sc2A (Fig. 5c), although above-median performance was achieved (Fig. 5d,e). These results show that the tested ensemble methods could match or modestly improve performance when the best algorithm was not known but at substantial computational costs (Supplementary Note 3).

## Discussion

Cancer is an evolutionary process and subclonal reconstruction from tumor DNA sequencing has become a central way to quantify this process^3,31,37,38^. Subclonal reconstruction is a complex and multifaceted mathematical and algorithmic process, with multiple distinct components^19^. Despite rapid proliferation of new methodologies, there has been limited benchmarking or even surveys of the relative performance of many methods on a single dataset^3,10,18^. Furthermore, despite the clear value of multisample and single-cell sequencing strategies, clinical studies have almost exclusively eschewed these for pragmatic, cost-effective bulk short-read sequencing of index or metastatic lesions^39,40^. By contrast, the length of individual sequencing reads continues to grow and this continues to improve variant detection (and, subsequently, subclonal reconstruction) by improving both mapping accuracy and phasing.

We report a crowd-sourced, benchmarking of subclonal reconstruction algorithms for single-sample designs. Characteristics of experimental design (sequencing depth) and cancer types (mutation load, purity, copy number, etc.) influence accuracy, especially by influencing NRPCC^10^. These results highlight trends in the influence of the underlying copy-number states on CP estimation. Algorithms are limited in the number of subclones they can confidently detect at a given depth but resolution increases with NRPCC. Practitioners should consider optimizing NRPCC rather than read depth for single-sample subclonal reconstruction. Other features influence the scores in an algorithm-dependent fashion and the choice of algorithm is the major determinant of high-quality subclonal reconstruction.

The error profiles and algorithmic features of top-performing subclonal reconstruction methods are not strongly correlated. Nevertheless, ensemble approaches for subclonal reconstruction do not generally exceed performance of the best individual methods. This is quite different from other applications in cancer genomics, potentially reflecting the complexity of the technical and biological features that influence accuracy. Improved ensemble strategies might be required to combine multiple algorithms in ways that leverage the interactions between specific tumor features and algorithm performance. Because different algorithms are best at different subtasks of subclonal reconstruction, we provide online tools to help users choose the best algorithm for their dataset and question of interest (https://mtarabichi.shinyapps.io/smchet_results/).

A key opportunity for simulator improvement is improved modeling of different aspects of cancer evolution, such as ongoing branching evolution in terminal (leaf) subclones^16^, spatial effects and mutation calling error characteristics. Systematic benchmarking of subclonal CNA is greatly needed, given its strong influence on downstream analyses. Improved simulations will likely interact closely with specific SNV detection strategies, suggesting that algorithm development should focus jointly on these two key features. Single-cell WGS may help build benchmarking datasets complementary to simulations, using pseudo-bulk as the ground truth^41–43^ while accounting for technical variation. As read lengths increase, additional opportunities will arise to use mutation-to-mutation and mutation-to-SNP phasing, particularly in high-SNV-burden tumors. Incorporation of this signal may resolve ambiguous phylogenies and improve subclonal reconstruction. We did not systematically consider balanced structural variants, which are often drivers and were not incorporated by any algorithm evaluated. Benchmarks on realistic datasets are needed to improve algorithm development and application.

## Methods

### Tumor designs and simulations

We designed 51 realistic tumor tree topologies with underlying subclonal structure: 16 tumor trees were inspired by published phylogenies^13,23–28^, 25 were based on manually reconstructed PCAWG trees^22^ and 10 were special theoretical cases based on the highly curated PD4120 (ref. ^13^). Tumors from the literature and from the PCAWG study covered some of the most common cancer types (breast cancer, prostate cancer, lung cancer, colorectal cancer and leukemia) and other sometimes less represented cancer types (pancreatic cancer, sarcoma, kidney cancer, brain cancer, lymphoma, head and neck cancer and thyroid cancer) (Supplementary Table 1).

PCAWG manual tree building was performed using DPClust (version 2.1.0) and Battenberg (version 2.2.10)^13^ using the pigeon-hole principle and mutation-to-mutation phasing to constrain the possible tree topologies. When multiple tree topologies were possible, we picked one at random for the simulation, while balancing branching and linear topologies across the full set of simulated tumors.

Each node was associated with a CP, specific whole-chromosome copy-number events and a number of SNVs and SVs, as well as expected trinucleotide contexts, which were all taken as input by our simulator^19^.

As described previously^19^, we used a custom BAMSurgeon^19,21^ pipeline (implemented in Perl version 5.26.3) to simulate BAM files with underlying tree topology and subclonal structure for the 51 tumors. Briefly, we began by aligning a high-depth (300×) Illumina paired-end publicly available BAM file (Genome in a Bottle GM24385) that was part of a father, mother and son trio using bwa (version 0.7.10) and the hs37d5 human reference. Following a standard variant-calling pipeline, we phased reads using PhaseTools (version 1.0.0)^19^, achieving a median phased contig length of ~85 kb. We then partitioned each phase and chromosome sub-BAM to simulate subclonal structure, adjusting the depth of each read pool by its CP and total fractional copies (that is, to simulate chromosome-length CNAs). We then spiked in SNVs, SVs and indels into each read pool using BAMSurgeon (version 1.2) while preserving phylogenetic ordering (thus, except for deletion events, a child subclone would contain its parent’s mutations). SNVs were distributed semirandomly to follow prespecified trinucleotide signatures and replication timing biases. We then merged sub-BAMs across phase and chromosome to obtain the final tumor BAMs. To obtain realistic SNV calls and copy-number profiles, MuTect (version 1.1.5)^29^ and Battenberg (version 2.2.10)^13^ were run on the simulated tumor and normal BAM files.

Battenberg was run to identify clonal and subclonal copy-number changes. Battenberg segments the mirrored B allele frequencies (BAFs) of phased heterozygous SNPs identified in the normal germline sample. It then selects a combination of purity and ploidy that best aligns the data to integer copy-number values in the tumor, akin to the allele-specific copy-number analysis of tumors (ASCAT)^46^. Finally, it infers mixtures of up to two allele-specific copy-number states from the BAF and log *R* of the obtained segments^13^. We compared the purity and ploidy values to the expected values from the designs and refitted the profiles if they did not agree. For this, we constrained the copy-number state of a clonally aberrated chromosome to its known design state. Reversing ASCAT’s equations, we could infer ploidy and purity from a given chromosome’s BAF and log *R* and derive the profile using the new pair of ploidy and purity values. Estimated purity values were expected to closely match the design exceptin special cases breaking the assumptions, especially those harboring a subclonal whole-genome doubling such as PD4120. Algorithms were run and scored on tumor VCFs and Battenberg outputs that excluded the X and Y chromosomes. Algorithms were allowed to run for up to 21 days on a compute node with at least 24 CPUs and 200 GB of RAM.

### Scoring metrics

For each subchallenge, we used different metrics that respected a set of criteria, as previously described^19^. These metrics are summarized below.

$${\rm{sc}}1{\rm{A}}=1-|\rho -{c}|$$

where *ρ* is the true cellularity, *c* is the predicted cellularity and |*x*| is the absolute value of *x*. Note that we require that 0 ≤ *ρ* ≤ 1 and 0 ≤ *c* ≤ 1.

$${\rm{sc}}1{\rm{B}}=[{L}-{d}+1]/({L}+1)$$

where *L* ≥ 1 is the true number of subclonal lineages, *d* is the absolute difference between the predicted and actual number of lineages, *d* = min(|*κ* − *L*|, *L* + 1). We do not allow *d* to be higher than *L* + 1 so that the SC1B score is always ≥0.

$${\rm{sc}}1{\rm{C}}=1-{\rm{EMD}}$$

where EMD is the normalized earth mover’s distance.

$${\rm{sc}}2{\rm{AB}}=\frac{\rm{AUPR}+\rm{AJSD}}{2}$$

where AUPR is the normalized area under the precision recall curve and AJSD is the normalized average Jensen–Shannon divergence. We normalize AUPR and AJSD by the worst AUPR and AJSD obtained by two extreme methods: assigning all SNVs to one cluster and assigning each SNV to its own cluster. sc2A takes the hard assignments, whereas sc2B takes the soft-assignment matrix.

$${\rm{sc}}3{\rm{AB}}={\rm{PCC}}$$

where PCC is the Pearson correlation coefficient between the predicted and true values from the coclustering matrix, cousin matrix, ancestor descendant matrix and the transposed ancestor descendant matrix. sc3A takes the hard assignments, whereas sc3B takes the soft-assignment matrix.

### Scoring and ranking

We scored outputs obtained from participant-submitted Dockerized Galaxy workflows using a Python (version 2.7.18) implementation of the scores described above (https://github.com/uclahs-cds/tool-SMCHet-scoring). Algorithm outputs were scored against truth files based on perfect SNV calls that contained all SNVs spiked in each tumor. False negatives were added to sc1C, sc2A, sc2B, sc3A and sc3B outputs as a single cluster with a CP of zero that was derived from the normal. False positives were excluded from outputs before scoring. We normalized the score *s* within each tumor and subchallenge across methods using min–max normalization (that is, offsetting and scaling such that the lowest and highest scores were set to 0 and 1, respectively).

$${s}_{i}^{\rm{minmax}}=\frac{{s}_{i}-{\rm{min}}(s)}{{\rm{max}}(s)-{\rm{min}}(s)}$$

where ${{s}}_{\rm{i}}^{{\rm{minmax}}}$ and *s*_*i*_ are the min–max normalized score and raw score of method *i*, respectively. We normalized the titration-series tumors simultaneously across all depths for a given tumor.

We ranked algorithms by normalized score across the 51 SMC-Het tumors, assigning any tied algorithms equal ranks. The best methods were defined as those with the highest median score across all tumors for which they produced a valid output.

As missing data could have been caused by technical restrictions that may not apply to users (for example, users would typically downsample SNVs in SNV-dense tumors) and the correct penalty for missing data is subjective, we did not penalize missing outputs. However, interested users can assign scores of zero to missing outputs in the interactive app and explore how they impact algorithm rankings (https://mtarabichi.shinyapps.io/smchet_results/).

### Random methods

For sc1A, we drew a single number from a uniform distribution between 0.2 and 0.99. For sc1B, we drew from four integer values {1, 2, 3, 4} with probabilities {0.2, 0.3, 0.3, 0.2}, respectively. For sc1C, we assigned one cluster to cancer cell fraction (CCF) 1 and, if there were multiple clusters, we assigned random CCF values to the other clusters by drawing from a uniform distribution between 0.2 and 0.9. We then assigned a random number of SNVs to each CCF cluster by drawing uniformly from 1 to 10. For sc2A, we assigned a proportion of SNV per cluster by drawing uniformly from 1 to 10 for each cluster. We then randomly assigned classes to SNVs. For sc2B, we generated 100 random vectors of SNV assignment to subclones and ran the function comp.psm from the R package mcclust (version 1.0) to obtain the proportions of coclustering.

### Linear models for tumor and algorithm features

All statistical analyses were performed in R (version 3.5). For each subchallenge, we first removed algorithms from the same team with scores that were highly correlated across tumors (*r* > 0.75), retaining the algorithm with the highest median score for each subchallenge. We derived 22 features to describe each tumor. Key features were defined as follows:

$$\rm PGA=\frac{Bases\,within\,CNAs}{Total\,bases\,in\,genome}$$

where CNAs were defined as segments within the Battenberg output where total clonal or subclonal copy number deviated from the integer tumor ploidy.

$${\rm{CF}}=\frac{\,m\,{\rm{in}}\,{\rm{clonal}}\,{\rm{node}}}{{\rm{Total}}\,m}$$

where *m* is the count of SNV, indels or SVs.

$${\rm{NPRCC}}=\frac{\rho d}{\rho \varPsi +2(1-\rho )}$$

where *d* is the read depth, *ρ* is the purity and *Ѱ* is the tumor ploidy.

Peak overlap was calculated by fitting density curves to each subclone in CCF space after adjusting each tumor’s VAF using true CNAs and CPs. To compute the relative proportion of CCF space covered by multiple subclones (peak overlap), we calculated the area underneath multiple CCF density curves relative to the total area as approximating integrals using the trapezoidal rule for each tumor. SNV, indel and SV counts were derived from the ground-truth files used to generate each tumor.

We collected algorithm features from teams through an online form filled at the time of algorithm submission into the challenge. For each algorithm feature within each subchallenge, we removed levels represented by fewer than three algorithms, as well as any level labeled ‘other’, to enhance model integrity and interpretability.

We then assessed the impact of tumor and algorithm features on scores using β regressions with the R package betareg (version 3.2) with a logit link function for the mean and an identity link function for *Ѱ* (which models variance) with only an intercept term^47^. We analyzed only sc1A, sc1C, sc2A and sc2B with β regressions as scores for sc1B were discrete proportions (difference between the true and predicted subclone number relative to the true subclone number) and measures of variance explained from binomial GLMs would not have been directly comparable. Effect size interpretation is similar to that of a logistic regression, representing a one-unit change in the log ratio of the expected score relative to its distance from a perfect score (that is, *β*_*x*_ = log(score/(1 − score)). Because they represent a change to a log ratio, the predicted change on a linear scale will depend on the reference score (see Fig. 3b for an example of effect size visualizations on a linear scale). We ran univariate models with only tumor features when we considered only the top five algorithms in each subchallenge (Fig. 3a), as well as models that included both tumor and algorithm features when we considered all algorithms that ranked above the one-cluster solution in a given subchallenge (Extended Data Fig. 5c). We used the same procedure to assess feature associations when controlling to algorithm ID. For these analyses, we excluded corner-case tumors and two tumors with >100,000 SNVs (P2 and P7) where only five algorithms produced outputs.

### Linear models for error bias

Bias in purity was assessed by taking the difference between the predicted and true purity for each tumor. We modeled inverse normal transformed errors using a linear regression that allowed interactions between NRPCC and algorithm ID in both the titration-series and the SMC-Het tumors (excluding corner cases). As the SMC-Het tumors contained two lower-NRPCC tumors, we verified that results remained consistent in their absence. We then extended this analysis to multivariate modeling with elastic net regressions as implemented in glmnet (version 2.0-18). Models were trained and assessed using nested cross-validation where one tumor was held out in each fold. We tuned *λ* and *α* in the inner loop and retained the value that achieved the lowest root-mean-squared error across the held-out samples. In each fold, we also removed features that were >70% correlated. We used the same framework on the full dataset to train the final model. We computed *R*^*2*^ on the basis of predictions in the held-out samples of the outer loop to estimate predictive performance.

We similarly analyzed the difference between the predicted and true number of subclones. For statistical modeling, we included only observations where error < 8 to minimize the effect of outliers and used a cumulative link ordinal regression implemented in MASS (version 7.3-51.6) to model the effect of NRPCC on subclone number estimation error when controlling for algorithm ID. We extended these to multivariate models using *L*^1^-regularized ordinal regression as implemented in ordinalNet (version 2.9). We trained and assessed these models using leave-one-tumor-out cross-validation. One tumor was held out in each fold and *R*^2^ was computed from correlating model predictions to the held-out tumors. Within each fold, we removed strongly correlated features (*r* > 0.7) and *λ* was tuned using the Akaike information criterion. We report effect sizes from the final model that was trained on the full dataset. We repeated both the purity estimation error and the subclone number estimation error multivariate analysis with and without algorithm ID terms. Effect sizes were congruent for both models but *R*^2^ decreased without algorithm ID terms.

### Genomic feature models

True CNA status was called on the basis of the known truth. If a region experienced both clonal and subclonal CNAs, then CNAs were labeled subclonal. Genomic features were extracted from the MuTect (version 1.1.5) VCF files using the Variant Annotation R package and from BAM files using Rsamtools (version 1.34.1) and bam-readcount (commit 625eea2). We modeled clonal accuracy using β regressions as described above. SNV CP error was modeled using linear regressions following an inverse normal transform. We excluded the corner-case tumors from all modeling unless stated otherwise.

### Battenberg assessment

For assessing Battenberg accuracy, Battenberg copy-number calls were obtained from the first solution provided in the Battenberg outputs. If a region was represented by multiple segments, we weighed each segment by its relative length and averaged its copy-number estimates. We considered a clonal CNA to be correct if the total copy number for the segment matched the total true copy number of the region. Similarly, a subclonal copy-number event was correct if Battenberg provided a clonal and subclonal copy-number solution (*P* < 0.05) and the total copy number matched the true copy number of any of the tumor leaf clones (for example clones that did not have children). We trained and assessed the *L*^1^-regularized logistic regression for correct Battenberg CNA calls using nested cross-validation as described above, tuning *λ* using the inner loop. As the dataset was highly unbalanced, within each fold, we sampled 250 CNAs where Battenberg was correct and included all 104 CNAs where Battenberg was incorrect, we resampled the latter through replacement with an additional 50 incorrect CNAs. Within each fold, we removed correlated features (*r* > 0.7) and optimized *λ* for sensitivity in the held-out samples. We repeated this procedure on the full dataset to train the final model.

### Neutral tail simulation and analysis

To quantify the impact of branching or neutral tail mutations on benchmark results and algorithm error profiles, we leveraged the simulation code on the basis of branching processes described by Tarabichi et al.^34^. We then modified this framework to expand subclones in silico that matched our predesigned phylogenies, while tracking all mutations at the single-cell level. We applied this to four of the five titration-series tumors (that is, tumors present at different average read coverage levels) reported previously by Salcedo et al.^19^. We simulated the growth of each tumor with four increasing mutation rates (mult1 = 5, mult2 = 10, mult5 = 25 and mult10 = 50 mutations per cell per division), effectively adjusting the relative number of tail mutations. The mutation rates aimed to cover a realistic but high range. We then modified the somatic SNV VCFs for each titration-series tumor to include both ‘neutral tail mutations’ and mutations appearing between subclonal generations (that is, those not present in the most recent common ancestor of the subclones but in all ancestors from divisions before and after its emergence).

These three steps yielded 80 new somatic SNV VCF files including tail and branching mutations. Because these were not read-level simulations but rather based on simulated read counts, we replicated mutation calling by retaining SNVs with an alternate read count ≥ 3. This strategy did not increase the number of false-positive somatic SNVs but accurately reflected the sensitivity of modern somatic SNV detection pipelines.

We then ran the top five algorithms for sc1A, sc1B, sc1C and sc2A using the original, submitted Docker containers and the VCFs that included filtered neutral tail mutations. We scored algorithm outputs using our established framework as described above. We ranked algorithms on the basis of median scores of the titration-series tumors and compared them to ranks generated from the same set of tumors before adding tail mutations. We then systematically compared the effect of neutral tail mutations on scores, purity estimation, subclone number estimation and SNV CP prediction by directly matching outputs from a given algorithm, tumor and depth before and after adding neutral tail mutations. Finally, we ran MOBSTER on the neutral tail mutation VCFs using the default parameters to identify and filter tail mutations. We adjusted input VAFs for CNAs using dpclust3p (https://github.com/Wedge-lab/dpclust3p, commit a505664). We tested for the effect of neutral tail filtration on cluster number using proportional-odds ordered logistic regression and on scores using GLMs (binomial family for sc1B and β regression for sc1C) controlling for tumor ID, algorithm ID and depth.

### Ensemble subclonal reconstruction

We ran ensemble methods on the outputs of four subchallenges: sc1A, sc1B, sc1C and sc2A. For sc1A, the ensemble approach was the median of the outputs. For sc1B, it was the floor of the median. For sc1C, we ran WeMe^31^, which takes a weighted median of the CCF and the proportion of SNVs assigned to the CCF to construct a consensus location profile, while ignoring individual SNVs assignments. Consensus for sc2A was performed using CICC^31^, which takes the hard cluster assignment of each SNV to clusters and performs a hierarchical clustering on the coassignment distances across methods between mutations to identify SNVs that most often cluster together across methods. We ran these approaches on 39 tumors, excluding the special cases and the two tumors with the largest number of SNVs (P2 and P7), for which most algorithms did not provide any outputs. For an increasing number of input algorithms, we ran the ensemble approaches on all possible combinations of algorithms, except when the possible number of combinations was >200, in which case we randomly sampled 200 combinations without replacement.

### Scores across multiple subchallenges and multicriteria decision

Akin to the PROMETHEE methodology used in decision engineering for the subjective choice of alternatives based on a set of quantitative criteria^48^, we performed principal component analyses on the weighted means of the scores across tumors in the subchallenge dimensions, representing ~66% of the variance in the data. We projected methods and subchallenges in that space. A decision axis was also projected as a weighted mean of the scores across subchallenges. Projection of the methods onto that axis led to a method ranking. To assess the stability of the decision axis upon weight changes, we also showed a density area for the decision axis projection defined by 3,000 decision axes obtained after adding −50% to 50% changes drawn uniformly to the subchallenge weights. We also randomly assigned weights to tumors (200 times) and subchallenges (200 times) from uniform distributions and derived 40,000 independent rankings.

### Data visualization

Figures were generated using R (version 4.0.5), Boutros Lab Plotting General (version 6.0.0)^49^, lattice (version 0.20–41), latticeExtra (version 0.6–28), gridExtra (version 2.3) and Inkscape (version 1.0.2). Partial residual plots were generated with the effects package (version 4.2). Color palettes were generated using the RColorBrewer package (version 1.1–2).

### Reporting summary

Further information on research design is available in the Nature Portfolio Reporting Summary linked to this article.

## Online content

Any methods, additional references, Nature Portfolio reporting summaries, source data, extended data, supplementary information, acknowledgements, peer review information; details of author contributions and competing interests; and statements of data and code availability are available at 10.1038/s41587-024-02250-y.

## Supplementary Information

Supplementary Information Supplementary Notes 1–3, Figs. 1–6 and Tables 4 and 5.

Reporting Summary

Supplementary Tables 1–3 Supplementary Table 1: All scores. Supplementary Table 2: Tumor features. Supplementary Table 3: Entry features.

## Data Availability

BAM files are available from the EGA at EGAS00001002092. SNV, SV, CNA and indel calls and corresponding truth files are available at https://www.synapse.org/#!Synapse:syn2813581/files/. The normal BAM with spiked-in mutations is available at https://www.ebi.ac.uk/ena/browser/view/PRJEB52520. Human genome assembly hs37d5 was used as the reference. Scores are available for download at https://mtarabichi.shinyapps.io/smchet_results/.

## Code availability

Participant-submitted Docker containers are available from Synapse at https://www.synapse.org/#!Synapse:syn2813581/docker/. Galaxy workflows are available at https://github.com/smc-het-challenge/. BAMSurgeon (version 1.2) is available at https://github.com/adamewing/bamsurgeon. The framework for subclonal mutation simulation is available at http://search.cpan.org/~boutroslb/NGS-Tools-BAMSurgeon-v1.0.0/. The PhaseTools BAM phasing toolkit is available at https://github.com/mateidavid/phase-tools. The SMC-Het scoring framework is available at https://github.com/uclahs-cds/tool-SMCHet-scoring.
