# Supplementary material for: Crowd-sourced benchmarking of single-sample tumor subclonal reconstruction
Source: Nat Biotechnol. 2024 Jun 11;43(4):581–92. doi: 10.1038/s41587-024-02250-y (PMC11994449; doi:10.1038/s41587-024-02250-y)
Supplement: Supplementary file 1 — Supplementary Notes 1–3, Figs. 1–6 and Tables 4 and 5. [file 41587_2024_2250_MOESM1_ESM.pdf]

nature biotechnology

Supplementary information https://doi.org/10.1038/s41587-024-02250-y

Crowd-sourced benchmarking of single-sample tumor subclonal reconstruction

In the format provided by the authors and unedited

Supplementary Information

Supplementary Note 1: Unpublished Winning Algorithm Descriptions 2

Supplemental Note 2: Branching Tails and the Benchmarking of Subclonal Reconstruction Algorithms 3

Supplemental Note 3: Decision theory - effects of the weights of criteria and of ensemble models on algorithm performance 9

Supplementary Figures

- Supplementary Figure 1. True tumour designs 10
- Supplementary Figure 2. Phylogeny inference assessment 11
- Supplementary Figure 3. Profiling tumour difficulty and feature associations with score 13
- Supplementary Figure 4. SNV cellular prevalence error profiling 15
- Supplementary Figure 5. Effect of neutral tail filtration on subclonal reconstruction 16
- Supplementary Figure 6. Variance of ensemble performance as function of number of input algorithms 17

Supplementary Tables

- Supplementary Table 4 18
- Supplementary Table 5 19
- Supplementary Table 6 20

Supplementary Note 1: Unpublished Winning Algorithm Descriptions

GISL. We developed a cascade ensemble model based on the Dirichlet process mixture model for tumour subclonal reconstruction. This model consists of four connected modules, named Module 1 (M1) to Module 4 (M4). Module M1 derives an initial estimate of cellularity from the phased phenotype information provided in the input Battenberg CNA data. This value is used in the subsequent steps to improve the accuracy of the decomposition results. Modules M2 and M3 predict the tumour subclonality based on a truncated Dirichlet process mixture model implemented by the blocked Gibbs sampler, but using different subsets of mutations. On the one hand, to consider the effect of CNAs, we perform the decomposition on a selected subset of mutations in M2 for which the total copy number exactly equals one. The expected variant allele frequencies for these mutation loci are deterministic and hence no arbitrary assumptions are needed. On the other hand, to reduce the effect of false positive mutation callings, we designed several filtering criteria making use of the information available in the input MuTect VCF data. Module M3 then performs the decomposition on the subset of mutations passing those filters. The above methods address the questions in SubChallenges 1 to 3. For SubChallenge 4, module M4 reconstructs the evolutionary relationships of the inferred subclones using a heuristic tree building method based on three assumptions: (a) infinite site (b) parsimony, and (c) that each subclone has no more than two child nodes.

This model has several advantages. First, the cascade ensemble architecture provides flexibility to customize for different practical application scenarios. Second, the core technique for inferring tumour subclones (modified truncated Dirichlet process mixture model) features the automatic generation of the number of components. Finally, during the subclonal reconstruction process, the method considers the effects from CNAs and the false positive mutation callings, and accordingly adjusts the predictions for improved accuracy.

Object Integration. This method relies solely on the Battenberg copy-number profiles to re-estimate the purity. It first identifies segments that are not flagged as subclonal by Battenberg, and derives separate purity estimates from both the BAF and LogR. From the fitted integer values and the BAF and LogR, it is possible to back-calculate the purity for each segment. Object Integration summarizes these estimates using linear modeling and iterative fitting and the length of the segments as weights. It then takes a median between those independent purity estimates if the range is lower than 0.1 or takes the highest purity estimate if not.

Supplemental Note 2: Branching Tails and the Benchmarking of Subclonal Reconstruction Algorithms

Introduction

Tumours evolve from normal cells which have acquired random somatic mutations throughout their lifetime[1]. Most are neutral and some may confer a selective advantage to their descendants. Over time this leads to a patchwork of selected and drifting clones making up normal healthy tissues. Subclonal reconstruction is the process of assigning mutations (genotypes) to ancestors from subpopulations of cells in a given sample by leveraging the shared allele frequencies of these mutations[2]. This is independent and agnostic of any relative fitness advantage or disadvantage of these subpopulations.

Subclonal divergence can occur through a variety of mechanisms. In a simple example, spatially-independent tumour sites that lack cell-to-cell exchange would diverge over time. This divergence could occur as a result of differences in the selective pressures experienced in the two different sites. These selective pressures could themselves change over time: e.g. one site might become hypoxic, and another not. But even in the absence of differential selective pressures, the two sites would diverge because of neutral evolution: random genetic drift in the face of mutations that do not lead to fitness differences.

Neutral evolution is ongoing in each cancer subclone -- whether or not it is experiencing a selective pressure. It occurs when cells divide and accumulate somatic mutations, most of which have little or no impact on cell fitness. Thus within a population, there is an increasing monotonic relationship between the inverse of allele frequencies and the number of mutations. These low-frequency mutations are often termed “branching” or “tail” or “neutral tail” mutations[3-6]. Identifying and accounting for branching mutations is important for studying tumour evolution as they can make up a large proportion of the observed mutations in certain tumours[6,7,8].

In practice, low-frequency mutations observed in modern DNA sequencing are a complex mixture of artefacts and true tail mutations[9]. DNA mutation detection is systematically more error-prone when the number of supporting reads is low -- and these errors include both false positives and false-negatives. At typical sequencing depths of 80x, a small number of tail mutations will be represented by even three reads[10]. However as sequencing depth increases into the low hundreds, and eventually into the thousands, the number detected will inexorably increase.

Because existing tail mutations are a mixture of branching true positives and low-allele-frequency false negatives, many groups elect to simply exclude low-CCF or low-read-support variants with hard-filters. An alternative and more sophisticated approach is implemented in the MOBSTER algorithm which was developed by Caravagna and colleagues with the intent of explicitly identifying tail mutations within subclonal reconstruction[6]. It models the CCF space as a mixture of Beta distributions and one 1-over-f tail, showing that adding the 1-over-f distribution usually leads to better fit of the data[6].

As observed by Caravagna and colleagues, correctly handling branching tail mutations in the context of subclonal reconstruction is important and challenging. Although these mutations are not technically representing a single extra subclone, for typical algorithms which do not model these

explicitly, they may induce an extra subclonal cluster to which the mutations at low frequencies could be assigned. This extra cluster in turn might "pull" mutations from true higher-frequency clusters.

The importance of branching tail mutations in subclonal reconstruction has been a topic of significant discussion over the past decade[7,3,4,6,11,12]. While most algorithms do not take tail mutations into account, some have suggested that they serve as a major confounder and systematically bias the performance of many methods[6]. This study does not aim to resolve this question, but here, we would like to address the impact that branching tail mutations may have on benchmarking.

Ground Truth

One question that any consideration of branching tails raises is that of ground truth. What exactly is the ground truth for any specific cancer? Our approach was to either observe or define a tumour phylogeny. This was sometimes done from tumours from the PCAWG consortium (n=25), sometimes from literature-reported phylogenies (n=16), and sometimes by updating a single base tumour to test important edge cases (e.g. germline mosaicism, over-dispersion, very low CCF, etc. -- these are outlined in **Extended Data Figure 1**). In each of these cases, what we used from the public data was the structure of the phylogenetic tree, the approximate number of somatic SNVs and CNAs, and the estimated tumour purity. These parameters were the only information taken from public data.

At that point, we took a very high-coverage normal sample (a Genome in a Bottle reference sample). We then directly simulated an evolutionarily accurate mutational profile from that normal reference. We took the full set of reads, and sampled the appropriate numbers required for both contaminating normal cells (e.g. for tumour purity simulation) and for correctly representing each subclone and CNA. We added appropriate numbers of somatic SNVs, reflecting both mutational timing and trinucleotide signatures, as well as SVs, using BAMSurgeon[9,13]. This yielded fully simulated tumours that derive from a normal reference, and share no specific mutations or read-level information with the original public tumours. Thus the only information used to design them from real tumours was: (i) purity, (ii) number of subclones, their CCFs and branching or linear relationship, (iii) number of clonal and subclonal CNAs, (iv) number of SNVs per subclone, and (v) proportions of detected trinucleotide signatures.

This extensive simulation framework has been previously reported and validated, first for somatic SNVs[13], then for somatic SVs[14] and finally for subclonal populations[9]. Thus its use here in this Challenge builds upon those prior validation studies and subsequent community usage of both the tooling and downstream simulated benchmark tumours reported therein. In the context of this report, then, the 51 designed tumours do indeed have a ground truth -- the specific tumour designs we simulated. These are reported in **Supplementary Figure 1** and **Extended Data Figure 1**, and reflect specific parameters reported in a range of publications and by a range of groups from different analytical methods with different considerations of branching tails.

We had considered potential circularity at length during our initial design and analysis. Several lines of evidence suggest that there was either little or no effect relative to the variability amongst tumours and methods, and that it did not influence the results reported here:

Participants were unaware of our tumour design strategy prior to their submission of Docker containers, so could not modify their methods, parameterizations or pipelines in response to this information.

We performed a manual review of all phylogenies to ensure no artefacts were present, particularly those modelled upon PCAWG tumours. This included manual use of the pigeonhole principle and additional mutation-to-mutation phasing to constrain the set of possible tree topologies. When multiple topologies were consistent with the data, we selected randomly, balancing branching *vs.* linear across the full set of tumours.

While the phylogenies were directly learnt from real tumours, only these hyper-parameters were retained. All individual somatic mutations were directly simulated at the read-level onto a consistent high-coverage germline sample (Genome in a Bottle, GM24385) and subjected to consistent somatic mutation detection pipelines. Thus, both the germline and somatic variants are not preserved, only the underlying parameters.

While 25 tumour phylogenies were derived from PCAWG, 16 others were not (those labelled T0 through T15). Across the 1,324 submissions for subChallenge 1A analysed, the mean score for the 25 PCAWG-derived tumours was 0.893 while that for the 16 non-PCAWG-derived tumours was 0.889. Thus it does not appear that across the entirety of the Challenge the method of design impacted scores.

Although models using Dirichlet process models did generally perform very moderately better to those that did not (**Extended Data Figure 5c-e**), we found that the parameterization and context of these models was critical to performance. For example, there was no systematic difference between binomial noise models vs. beta-binomial noise models, or between fixed *vs.* latent concentration parameters. This held true for both PCAWG tumours and non-PCAWG ones, and there was no apparent bias for the methods used to generate the parameters we used to design the tumours.

Taken together, these data support the idea that there is no apparent bias introduced by our use of real tumour data in selecting design parameters for a subset of the tumours in this Challenge, particularly given the use of simulated read-level data that can accurately model many of the genomic artefacts experienced by modern subclonal reconstruction methods in real use.

Synthetic Tails

However, we also explicitly considered tail effects by using an agent-based model to inject them into tumours at different frequencies. We find that the size of these tails/false clusters and their pull are very limited at currently common sequencing depths (<128X). This is evidenced in several ways, including by the simulations at increasing mutation rates and the very limited effect on assignment scores (**Extended Data Figure 9**). At higher sequencing depths (>64x), the effect becomes increasingly noteworthy, and we imagine that at very high coverage (>500x), it would be quite substantial.

While the number of subclones inferred by most algorithms increases with the presence of tail mutations, it is also strongly influenced by technical parameters. The strongest of these is the number of reads per tumour chromosome copies (NRPCC)[10,15]. NRPCC dominates over other biological and technical factors in driving the inferred number of subclones, including branching tails. Thus one consequence of our study is to suggest significant caution when using the number

of subclones as a biological finding. What is important, stable and central to phylogenetic reconstruction is the correct assignment of mutations to their subclonal clusters.

Consistent with these findings, MOBSTER filtering, which identifies and removes tail mutations, can mitigate both false clusters and tail mutations. As one would expect, removing tail mutations significantly improves mutation assignment scores, especially as the branching tail size increases and at depth >64x. It also has the added benefit of removing many false positive mutations. Thus, pre-filtering could be incorporated into subclonal reconstruction pipelines when there is sufficient sequencing depth. The precise benefits of such filtering across a broad range of tumour and genomic contexts remain unclear, but this study suggests they may be worth defining, especially in the face of high-coverage sequencing and high NRPCC sequencing.

Summary

Cancer evolution is a very complex process, occurring in the context of a changing fitness landscape, and partial spatial separation of populations. Cancer cell fitness is influenced by both genetic and epigenetic phenomena. While ongoing neutral evolution plays a role in cancer evolution, its magnitude of importance in subclonal reconstruction studies is likely quite context-dependent. For example, at lower NRPCC values, almost no branching tail mutations will be detectable.

The debate around the importance of branching tail mutations will continue to progress with new data from deeper sequencing studies, from long-read sequencing, from multi-region sequencing, and from single-cell sequencing. In the context of this benchmarking effort, however, some conclusions can already be drawn. First, the presence of branching tails does not significantly change the relative ordering or error profiles of subclonal reconstruction algorithms, as the inter-method variability was larger than the variability associated with branching tails. Second, the impact of branching tails is context-dependent, affecting some aspects of subclonal reconstruction more than others. For example, deterministic clustering (sc2A) was more impacted than tumour purity estimation (sc1A) or subclone number (sc1B). Third, the impact of branching tails proceeded in the expected direction: to spuriously increase subclone numbers. Fourth, technical factors (most notably NRPCC) contribute significantly more to reconstruction accuracy than the presence *vs.* absence of branching tail. Fifth, removal of tails can clearly improve reconstructions in at least a subset of tumours.

Overall, our benchmarking confirms that branching tails influence tumour subclonal reconstruction, but also validated that other features can have very large effects. As sequencing technologies continue to mature, leading to increases in read-lengths, coverage (*i.e.* NRPCC), and the availability of multi-sample spatial and/or longitudinal sequencing, further evaluation of branching tails will be an important community need.

References

1. Martincorena, I. Somatic mutation and clonal expansions in human tissues. *Genome Medicine* **11**, 35 (2019).
2. Dentro, S. C., Wedge, D. C. & Van Loo, P. Principles of Reconstructing the Subclonal Architecture of Cancers. *Cold Spring Harb Perspect Med* **7**, (2017).
3. Williams, M. J., Werner, B., Barnes, C. P., Graham, T. A. & Sottoriva, A. Identification of neutral tumor evolution across cancer types. *Nat Genet* **48**, 238–244 (2016).
4. Williams, M. J. *et al.* Quantification of subclonal selection in cancer from bulk sequencing data. *Nature Genetics* 1 (2018) doi:10.1038/s41588-018-0128-6.
5. Davis, A., Gao, R. & Navin, N. Tumor evolution: Linear, branching, neutral or punctuated? *Biochimica et Biophysica Acta (BBA) - Reviews on Cancer* **1867**, 151–161 (2017).
6. Caravagna, G. *et al.* Subclonal reconstruction of tumors by using machine learning and population genetics. *Nature Genetics* **52**, 898–907 (2020).
7. Sottoriva, A. *et al.* A Big Bang model of human colorectal tumor growth. *Nat Genet* **47**, 209–216 (2015).
8. Sun, R. *et al.* Between-Region Genetic Divergence Reflects the Mode and Tempo of Tumor Evolution. *Nat Genet* **49**, 1015–1024 (2017).
9. Salcedo, A. *et al.* A community effort to create standards for evaluating tumor subclonal reconstruction. *Nat Biotechnol* **38**, 97–107 (2020).
10. Tarabichi, M. *et al.* A practical guide to cancer subclonal reconstruction from DNA sequencing. *Nature methods* **18**, 144–155 (2021).
11. Heide, T. *et al.* Reply to 'Neutral tumor evolution?' *Nature Genetics* **50**, 1633–1637 (2018).
12. Tarabichi, M. *et al.* Neutral tumor evolution? *Nat Genet* **50**, 1630–1633 (2018).
13. Ewing, A. D. *et al.* Combining tumor genome simulation with crowdsourcing to benchmark somatic single-nucleotide-variant detection. *Nat. Methods* **12**, 623–630 (2015).

14. Lee, A. Y. *et al.* Combining accurate tumor genome simulation with crowdsourcing to benchmark somatic structural variant detection. *Genome Biology* **19**, 188 (2018).
15. Dentro, S. C. *et al.* Characterizing genetic intra-tumor heterogeneity across 2,658 human cancer genomes. *Cell* **184**, 2239–2254 (2021).

Supplemental Note 3: Decision theory - effects of the weights of criteria and of ensemble models on algorithm performance

From a user's perspective, SubChallenge performance are criteria on which to base algorithm choice. Different use-cases might focus on the outputs from different SubChallenges. Moreover, the designs of some tumour phylogenies might be more representative for a given user's application, *e.g.* higher mutation load, lower chromosomal instability or higher purity. Therefore, the optimal algorithm choice depends on a specific use-case.

Figure 5a visualizes the decision space, simultaneously showing algorithm performance across SubChallenges (coordinate of each algorithm on each SubChallenge axis across tumours), dissimilarities across SubChallenges (angle between SubChallenge axes) and across algorithms (distance between algorithms). The blue "decision axis" shows the axis of average score across SubChallenges when all SubChallenges and tumours are weighted equally. In this case, it is stable to small fluctuations in these weights (shown by the decision "brane" around the decision axis; **Figure 5a**).

To better understand the sensitivity of algorithm selection to these weights, we simulated forty thousand "studies". In each, weights were randomly assigned to SubChallenges and tumours. The weighted average scores across SubChallenges and tumours was used to rank algorithms within each study. We performed simulations for three groups of SubChallenges: {sc1B, sc1C}, {sc1B, sc1C, sc2A} and {sc1B, sc1C, sc2A, sc2B} (**Figure 5b**). Across groups of SubChallenges, 12 algorithms (35%) reach a top rank within at least one study, while 22 (65%) were never ranked first. Users can adjust weights to reflect their use-cases in our dynamic web-applications (https://mtarabichi.shinyapps.io/smchet_results/) to facilitate algorithm choice.

Some users might have the resources to run multiple algorithms. Ensemble approaches have shown improved performance in other bioinformatics applications but are not mature for subclonal reconstruction and rarely applied. In this work we evaluated ensemble algorithms based on central tendency: the median of sc1A and the floor of the median for sc1B, and the ensemble methods recently developed by the PCAWG consortium[1] for sc1C and sc2A (**Methods**). We ran these ensemble methods for each SubChallenge on estimates from a large representative subset of all possible algorithm combinations (**Methods**) and for all tumours, after excluding the ten special cases and two tumours with over 100,000 SNVs for which only five algorithms produced outputs. The highest ensemble performance was reached with a low number of input algorithms (two for sc1A, four for sc1B, three for sc1C and three for sc2A). These results suggest that ensemble approaches, particularly those built from multiple top-performing methods, represent a robust algorithm choice in situations where the top-scoring algorithms for a specific dataset are not known, an ensemble of all algorithms would also result in robust solutions[1].

1. Dentro, S. C. *et al.* Characterizing genetic intra-tumor heterogeneity across 2,658 human cancer genomes. *Cell* **0**, (2021).

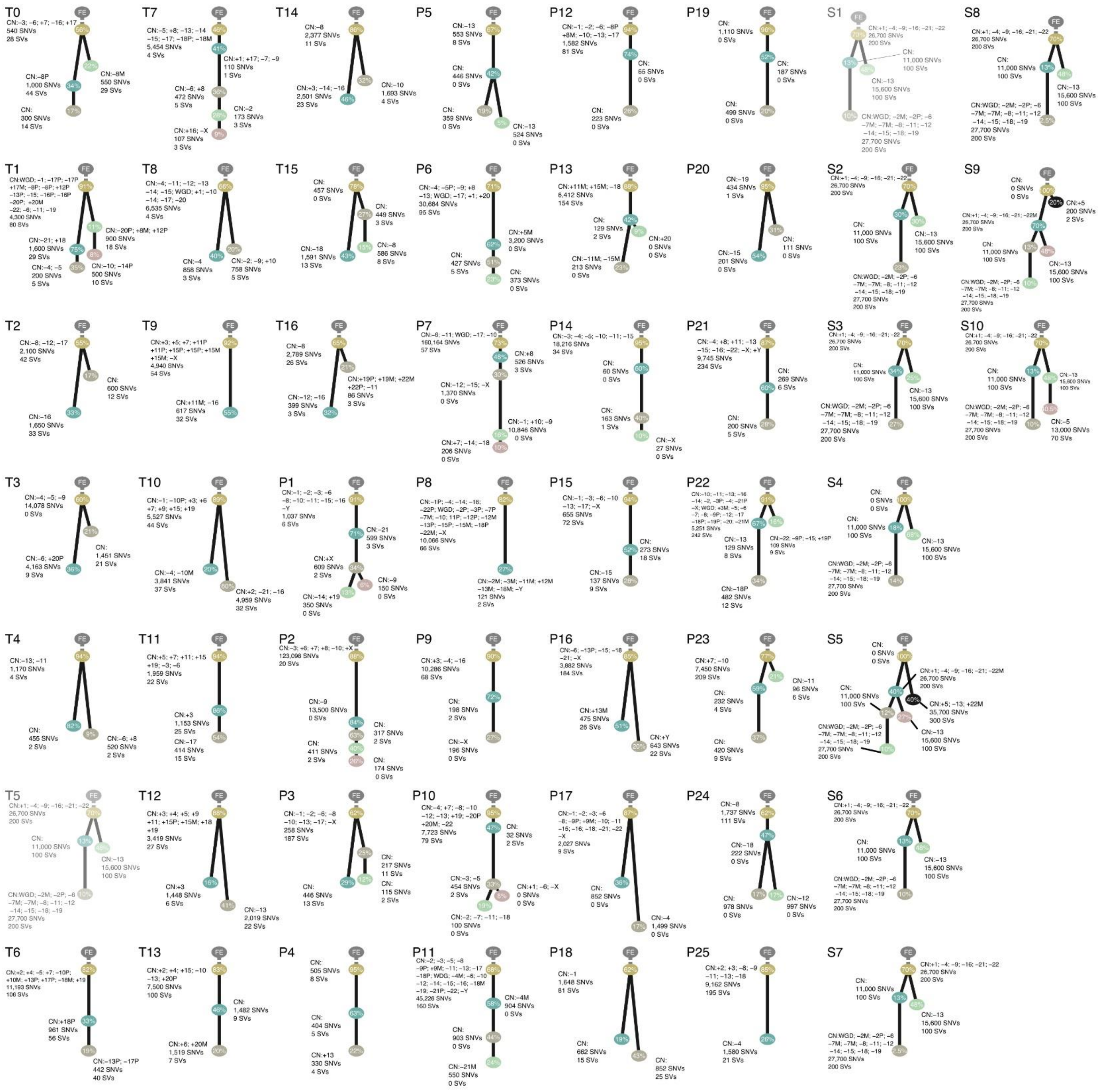

Supplementary Figure 1. True tumour designs

All 51 phylogenies originally designed for the challenge (52 trees are shown but T5 and S1 (shaded) are the same phylogeny based on PD4120 - this topology is both from the literature and a special case). From the fertilized egg (FE) to the first clone and subclones, we show cellular prevalences as percentages in the circles, next to which copy number events (CN) with losses (-) and gains (+) of whole chromosomes and whole-genome duplication (WGD) events are shown, along with total number of SNVs and SVs. The length of the branches is proportional to the number of SNVs.

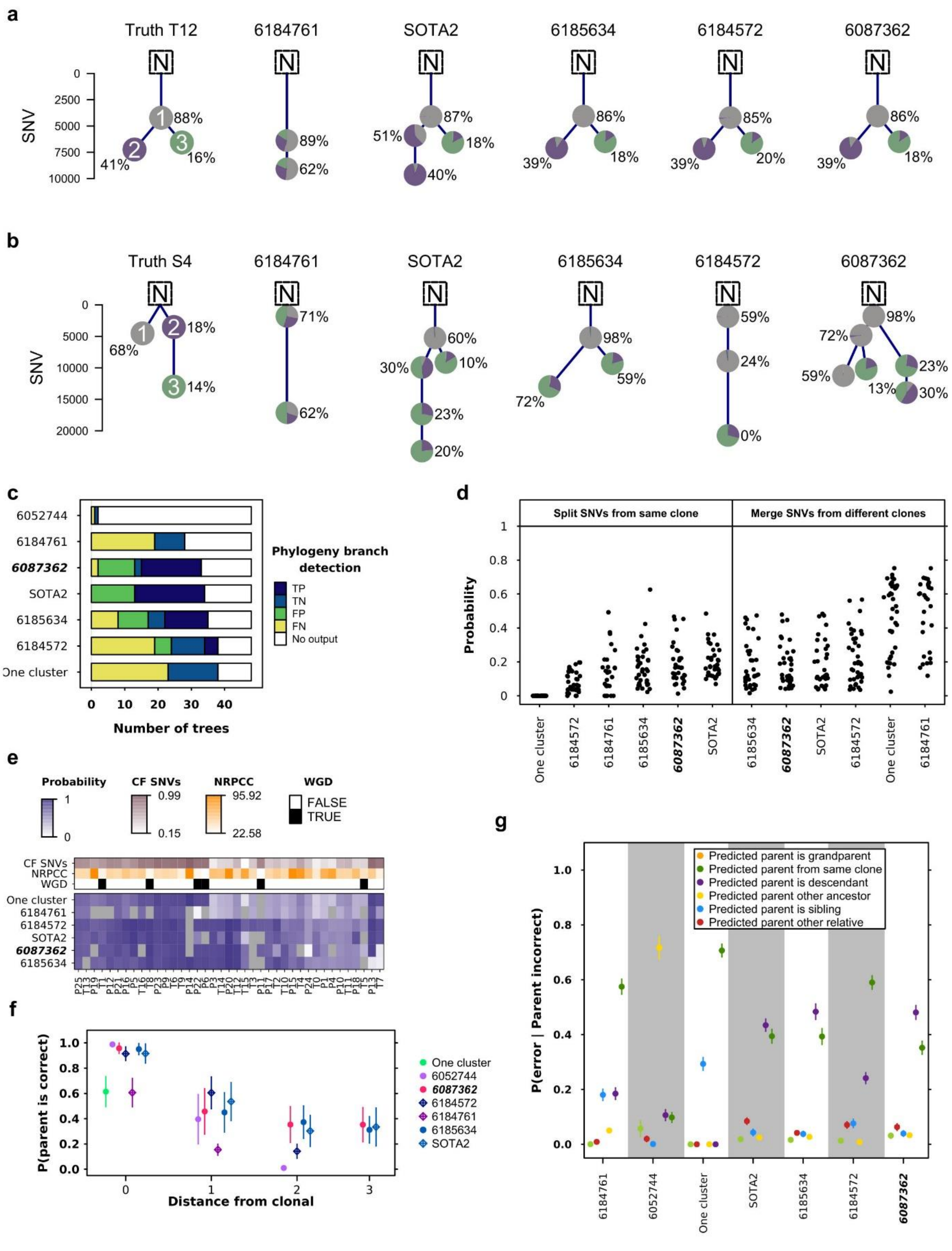

Supplementary Figure 2 Phylogeny inference assessment

a) Sample true (left) and predicted tree phylogenies for T12. Each node is annotated with its CP. Branch length is proportional to the number of SNVs in a given node and the area of each colour inside corresponds to the proportion of SNVs it contains from the corresponding true node. **b)** As

a) for the special case tumour S4 which had two clonal nodes. **c)** Each algorithm's error profile for detecting branching phylogenies (*e.g.* nonlinear phylogenetic trees). **d)** The probability that each SNV from the same clone in the true tree was predicted to be in a different clone (left) and the probability that two SNVs predicted to be in the same clone are actually from different ones in the true tree. Each point represents the probability from one tumour for a given algorithm (N=206 {tumour, algorithm} predictions). **e)** The probability that the predicted parent of a randomly drawn SNV is correct across all tumours, with tumour specific covariates shown in the top-most heatmap. **f)** The mean probability and standard error that the predicted parent for an SNV randomly drawn from a predicted tree is correct depending on its inferred relatedness to the clonal node (*i.e.* 0 indicates the SNV is within the predicted clonal node and 3 indicates the SNV is within a subclone that is the great-grandchild of the predicted clonal node). N=1529814 predicted SNV assignments. **g)** For an SNV randomly drawn from a predicted tree and a SNV randomly drawn from its predicted parent clone, the mean probability and standard error of each error case across tumours for each algorithm. N=1529814 predicted SNV assignments. Algorithms are ordered by median sc3A score and the top performing algorithm for SubChallenge sc3A is shown in bold italic text.

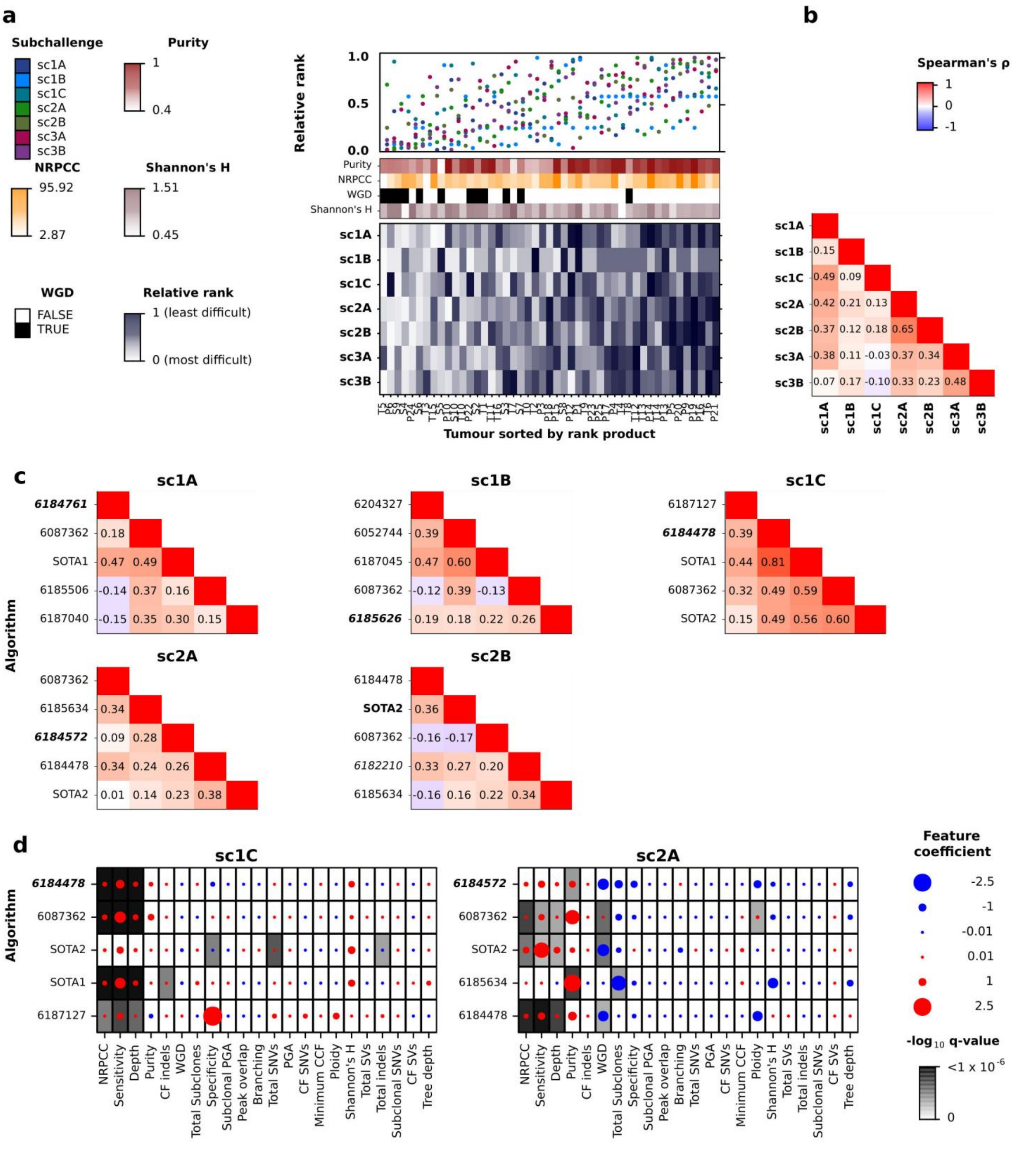

Supplementary Figure 3. Profiling tumour difficulty and feature associations with score

a) Tumour rank among SubChallenges, based on median score in a given SubChallenge. Bottom panel shows relative rank (rank/total number of tumours) for each tumour on each SubChallenge and the corresponding tumour features are shown above. Relative rank for each tumour on each SubChallenge is also shown in the top scatterplot. Tumours are ordered by rank product across

SubChallenges. **b)** Correlation for median tumour score among SubChallenges. **c)** Correlations in scores among the top five algorithms in each SubChallenge. The highest absolute correlation for each SubChallenge is shown. **d)** Results of univariate generalized linear models for tumour features on scores (β regression with a logit link) for each of the top five algorithms for sc1C and sc2A (sorted by ascending rank). The top-performing algorithms are highlighted in bold-italic. The size of the dots shows the feature effect size and the background colour shows the two-sided GLM Wald test P-value for feature coefficients after FDR adjustment. Effect size interpretation is similar to that of a logistic regression, representing a one unit change in the log ratio of the score relative to its distance from a perfect score (*i.e.* $\beta x = \log(\text{score}/(1-\text{score}))$).

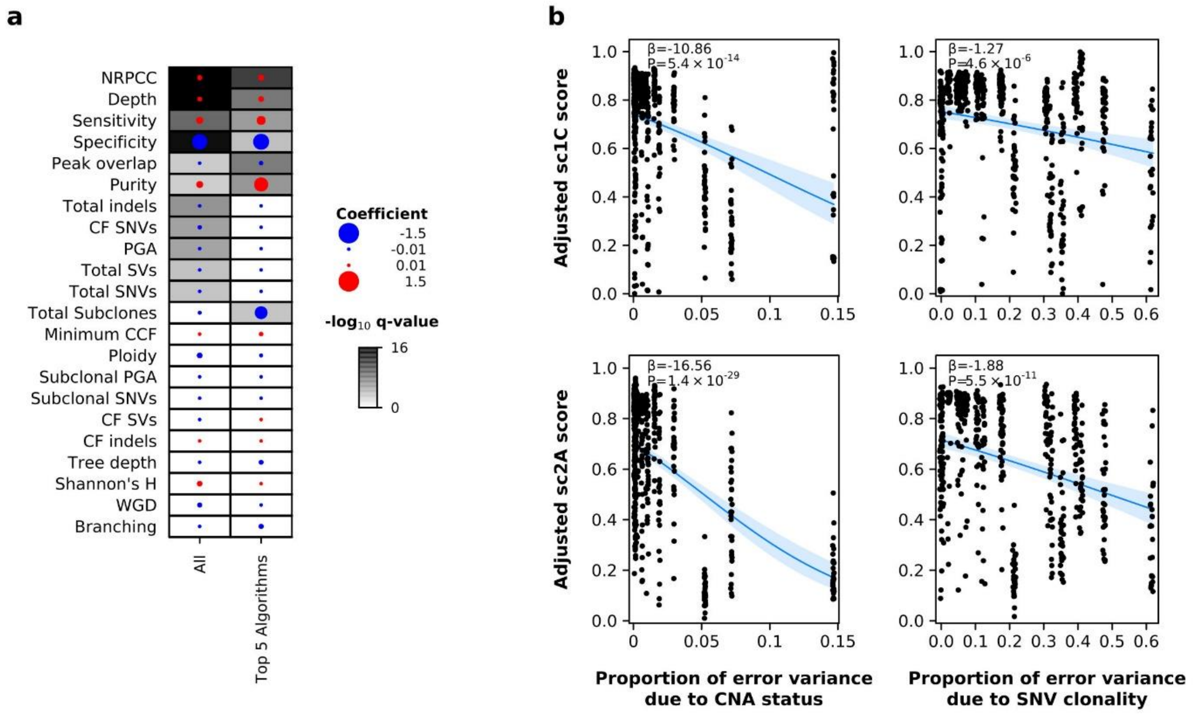

Supplementary Figure 4. SNV cellular prevalence error profiling

a) Results of generalized linear models for tumour features on clonal accuracy (β-regression with a logit link) that controlled for entry-ID. The size of the dots shows the effect size and the background colour shows the two-sided GLM Wald test P-value after Bonferroni adjustment. **b)** Partial residual plots showing the relationship between sc1C and sc2A scores after adjusting for covariates and the proportion of variance in SNV CP error explained by CNAs and SNV clonality for each algorithm specific model in **Figure 4e**. Lines show the mean effect averaged across all covariates and the shaded region shows the confidence interval

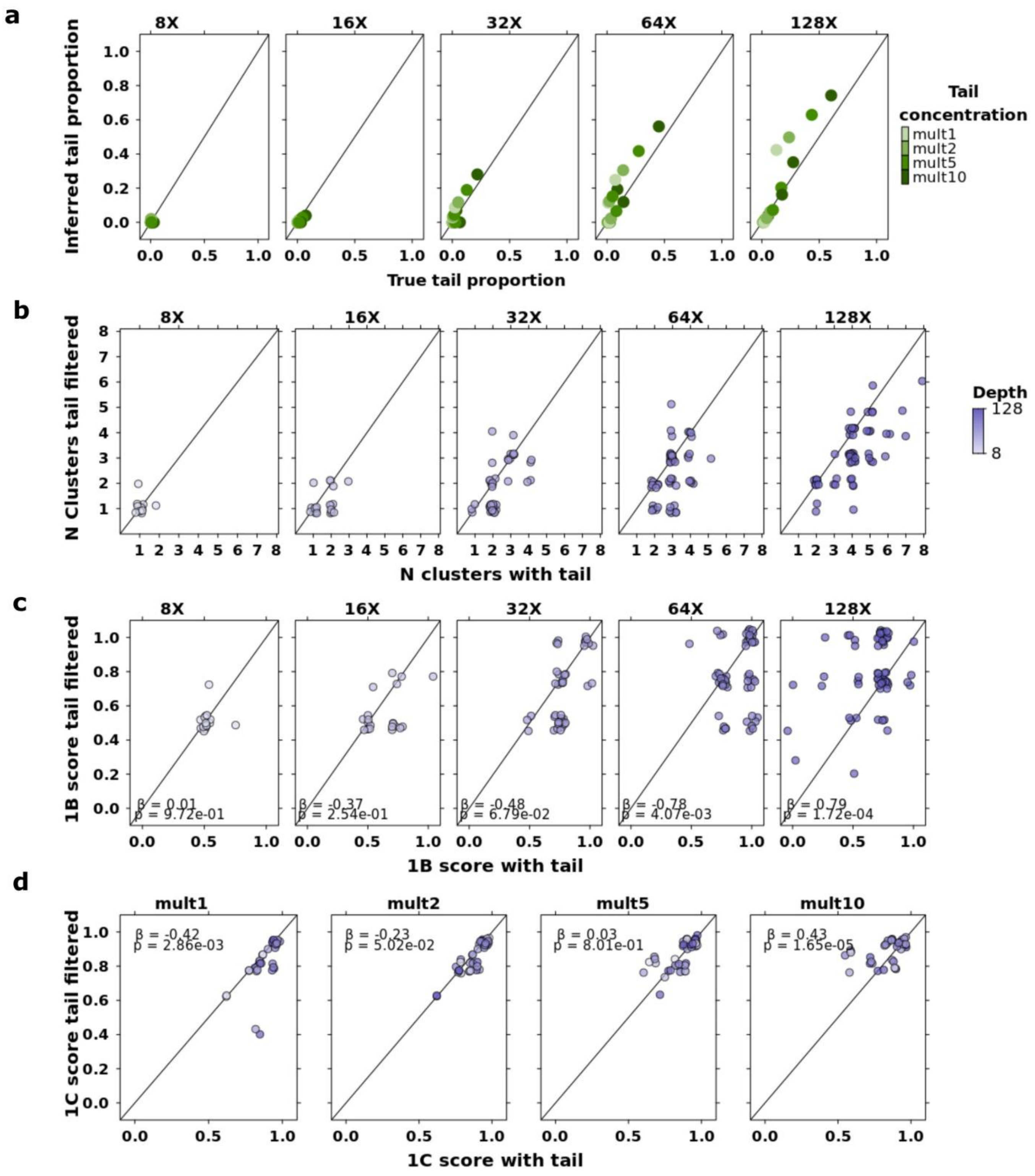

Supplementary Figure 5. Effect of neutral tail filtration on subclonal reconstruction

a) Comparison of the proportion of mutations attributed to a neutral tail by MOBSTER at each depth to the true proportion for the 39/80 VCFs where MOBSTER identified a neutral tail. **b)** Number of clusters identified through subclonal reconstruction before and after filtering neutral tail mutations. **c,d)** Score for 1B **(c)** and 1C **(d)** before and after filtering neutral tail mutations by depth and tail concentration, respectively. Effect size and two-sided Wald p-values from generalized linear models testing the effect of filtration on scores (controlling for tumour ID, algorithm ID and depth or neutral mutation rate) are shown.

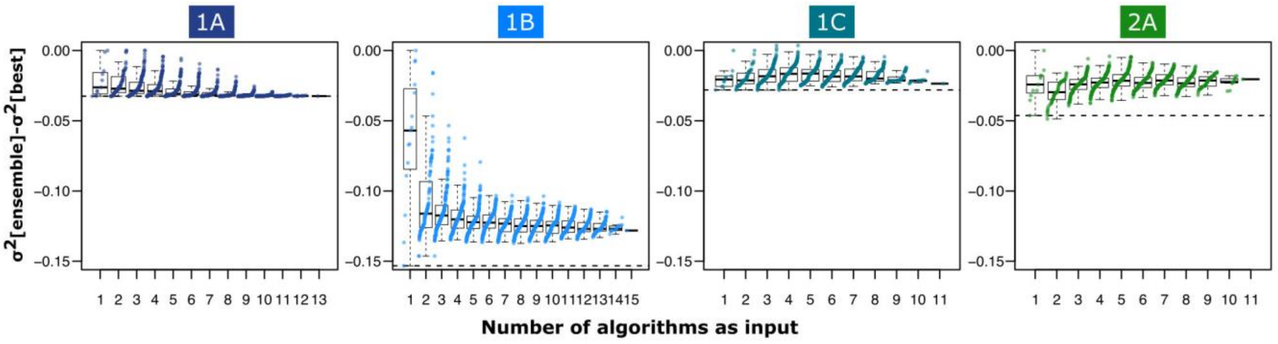

Supplementary Figure 6. Variance of ensemble performance as function of number of input algorithms

For sc1A, sc1B, sc1C and sc2A, we show the variance of the ensemble method minus the lowest variance of the individual methods (y-axis) as a function of the number of input methods (on the x-axis). Boxes extend from the 0.25 to the 0.75 quartile of the data range with a line showing the median. Whiskers extend to the furthest data point within 1.5 times the interquartile range. The dashed line represents the lowest variance of the individual methods. N= 1780 independent ensembles.

Supplementary Table 4: Regression results for clonal accuracy and SNV CP error. β regressions were used for accuracy and a linear model was used on inverse-normal transformed errors. Two-sided GLM Wald p-values are shown for accuracy and two-sided linear regression p-values are shown for errors. N=4968 and N=4462484, respectively.

	Estimate Accuracy	Std. Error Accuracy	P-value Accuracy	Estimate Error	Std. Error Error	P-value Error
(Intercept)	-2.06x10^{-1}	1.48x10^{-1}	1.63x10^{-1}	5.26x10^{-1}	3.81x10^{-3}	$<$ 2x10^{-16}
clonal loss	2.77x10^{-2}	7.59x10^{-2}	7.15x10^{-1}	1.98x10^{-1}	2.50x10^{-3}	$<$ 2x10^{-16}
clonal loss*SNV clonal	2.60x10^{-1}	1.05x10^{-1}	1.29x10^{-2}	4.50x10^{-2}	2.80x10^{-3}	$<$ 2x10^{-16}
clonal gain	1.21x10^{-1}	8.98x10^{-2}	1.76x10^{-1}	-1.53x10^{-1}	1.97x10^{-3}	$<$ 2x10^{-16}
clonal gain*SNV clonal	-5.33x10^{-2}	1.21x10^{-1}	6.60x10^{-1}	9.99x10^{-2}	2.06x10^{-3}	$<$ 2x10^{-16}
subclonal loss	6.55x10^{-2}	8.04x10^{-2}	4.15x10^{-1}	-2.13x10^{-2}	2.36x10^{-3}	$<$ 2x10^{-16}
subclonal loss*SNV clonal	-3.29x10^{-1}	1.11x10^{-1}	3.06x10^{-3}	-1.43x10^{-1}	2.75x10^{-3}	$<$ 2x10^{-16}
subclonal gain	-1.41x10^{-1}	8.91x10^{-2}	1.13x10^{-1}	2.09x10^{-1}	3.20x10^{-3}	$<$ 2x10^{-16}
subclonal gain*SNV clonal	3.18x10^{-1}	1.20x10^{-1}	7.98x10^{-3}	-2.03x10^{-1}	3.63x10^{-3}	$<$ 2x10^{-16}
SNV clonal	1.44x10^{-1}	7.28x10^{-2}	4.82x10^{-2}	-7.47x10^{-1}	1.16x10^{-3}	$<$ 2x10^{-16}
6052744	5.68x10^{-1}	1.17x10^{-1}	1.21x10^{-6}	-1.50	2.15x10^{-3}	$<$ 2x10^{-16}
6087270	6.59x10^{-1}	1.15x10^{-1}	9.77x10^{-9}	-1.37	2.06x10^{-3}	$<$ 2x10^{-16}
6087309	6.92x10^{-1}	1.20x10^{-1}	6.97x10^{-9}	-4.88x10^{-1}	2.32x10^{-3}	$<$ 2x10^{-16}
6087362	7.38x10^{-1}	1.15x10^{-1}	1.40x10^{-10}	-1.81x10^{-1}	2.05x10^{-3}	$<$ 2x10^{-16}
6181022	4.68x10^{-2}	1.16x10^{-1}	6.86x10^{-1}	-9.63x10^{-1}	2.00x10^{-3}	$<$ 2x10^{-16}
6184474	7.20x10^{-1}	1.14x10^{-1}	2.65x10^{-10}	-2.96x10^{-1}	2.00x10^{-3}	$<$ 2x10^{-16}
6184478	7.24x10^{-1}	1.14x10^{-1}	2.10x10^{-10}	-2.36x10^{-1}	2.00x10^{-3}	$<$ 2x10^{-16}
6184572	6.73x10^{-1}	1.15x10^{-1}	4.24x10^{-9}	-3.03x10^{-1}	2.04x10^{-3}	$<$ 2x10^{-16}
6184761	9.40x10^{-3}	1.20x10^{-1}	9.38x10^{-1}	3.98x10^{-1}	2.19x10^{-3}	$<$ 2x10^{-16}
6185634	7.97x10^{-1}	1.20x10^{-1}	3.02x10^{-11}	5.76x10^{-2}	2.31x10^{-3}	$<$ 2x10^{-16}
6187040	7.49x10^{-1}	1.22x10^{-1}	9.65x10^{-10}	-1.63x10^{-1}	2.36x10^{-3}	$<$ 2x10^{-16}
6187081	5.56x10^{-1}	1.19x10^{-1}	2.77x10^{-6}	-2.16x10^{-1}	2.11x10^{-3}	$<$ 2x10^{-16}
6203106	5.00x10^{-1}	1.29x10^{-1}	1.06x10^{-4}	-4.05x10^{-1}	2.37x10^{-3}	$<$ 2x10^{-16}
6400919	5.86x10^{-1}	1.15x10^{-1}	3.17x10^{-7}	-4.53x10^{-1}	2.00x10^{-3}	$<$ 2x10^{-16}
SOTA1	6.72x10^{-1}	1.15x10^{-1}	4.86x10^{-9}	-7.22x10^{-2}	2.06x10^{-3}	$<$ 2x10^{-16}
SOTA2	7.45x10^{-1}	1.15x10^{-1}	1.06x10^{-10}	-2.36x10^{-1}	2.19x10^{-3}	$<$ 2x10^{-16}
PCAWG-IR	5.55x10^{-1}	1.15x10^{-1}	1.33x10^{-6}	-1.15x10^{-1}	2.00x10^{-3}	$<$ 2x10^{-16}
6181088	7.41x10^{-1}	1.14x10^{-1}	7.48x10^{-11}	6.18x10^{-1}	2.00x10^{-3}	$<$ 2x10^{-16}
6613462	8.28x10^{-2}	1.24x10^{-1}	5.03x10^{-1}	-6.63x10^{-1}	2.11x10^{-3}	$<$ 2x10^{-16}
P10	-1.28x10^{-1}	1.50x10^{-1}	3.94x10^{-1}	-1.82x10^{-1}	3.98x10^{-3}	$<$ 2x10^{-16}
P11	-2.24x10^{-1}	2.02x10^{-1}	2.68x10^{-1}	5.07x10^{-1}	3.89x10^{-3}	$<$ 2x10^{-16}
P12	2.35x10^{-1}	1.88x10^{-1}	2.11x10^{-1}	4.37x10^{-2}	5.36x10^{-3}	3.47x10^{-16}
P13	4.92x10^{-1}	1.55x10^{-1}	1.52x10^{-3}	2.31x10^{-1}	4.14x10^{-3}	$<$ 2x10^{-16}
P14	1.75x10^{-1}	2.10x10^{-1}	4.04x10^{-1}	2.76x10^{-1}	3.84x10^{-3}	$<$ 2x10^{-16}
P15	3.81x10^{-1}	1.68x10^{-1}	2.34x10^{-2}	-6.73x10^{-2}	5.97x10^{-3}	$<$ 2x10^{-16}
P16	2.44x10^{-1}	1.67x10^{-1}	1.43x10^{-1}	1.90x10^{-1}	3.95x10^{-3}	$<$ 2x10^{-16}
P17	3.22x10^{-1}	1.64x10^{-1}	4.98x10^{-2}	1.11x10^{-1}	4.26x10^{-3}	$<$ 2x10^{-16}
P18	9.68x10^{-3}	1.91x10^{-1}	9.59x10^{-1}	5.78x10^{-2}	4.45x10^{-3}	$<$ 2x10^{-16}
P19	5.10x10^{-1}	2.59x10^{-1}	4.90x10^{-2}	2.02x10^{-2}	6.09x10^{-3}	9.16x10^{-4}
P20	5.62x10^{-1}	1.62x10^{-1}	5.36x10^{-4}	-1.26x10^{-1}	6.16x10^{-3}	$<$ 2x10^{-16}
P21	7.89x10^{-2}	1.69x10^{-1}	6.41x10^{-1}	2.03x10^{-1}	3.90x10^{-3}	$<$ 2x10^{-16}
P22	-7.42x10^{-4}	1.55x10^{-1}	9.96x10^{-1}	1.77x10^{-1}	4.55x10^{-3}	$<$ 2x10^{-16}
P23	1.25x10^{-2}	1.56x10^{-1}	9.36x10^{-1}	2.30x10^{-1}	4.00x10^{-3}	$<$ 2x10^{-16}
P24	1.61x10^{-1}	1.73x10^{-1}	3.51x10^{-1}	1.66x10^{-1}	4.61x10^{-3}	$<$ 2x10^{-16}
P25	3.69x10^{-1}	1.57x10^{-1}	1.89x10^{-2}	2.40x10^{-1}	3.83x10^{-3}	$<$ 2x10^{-16}
P3	3.16x10^{-1}	1.84x10^{-1}	8.67x10^{-2}	-7.09x10^{-2}	6.28x10^{-3}	$<$ 2x10^{-16}
P4	3.06x10^{-1}	1.95x10^{-1}	1.16x10^{-1}	-1.96x10^{-1}	6.27x10^{-3}	$<$ 2x10^{-16}
P5	3.98x10^{-1}	1.66x10^{-1}	1.67x10^{-2}	6.49x10^{-2}	4.45x10^{-3}	$<$ 2x10^{-16}
P6	-1.09x10^{-1}	2.20x10^{-1}	6.20x10^{-1}	4.99x10^{-1}	3.88x10^{-3}	$<$ 2x10^{-16}
P8	2.49x10^{-1}	1.48x10^{-1}	9.29x10^{-2}	2.67x10^{-1}	4.06x10^{-3}	$<$ 2x10^{-16}
P9	2.85x10^{-1}	1.68x10^{-1}	8.91x10^{-2}	2.29x10^{-1}	3.88x10^{-3}	$<$ 2x10^{-16}
T0	-7.65x10^{-2}	1.56x10^{-1}	6.24x10^{-1}	-1.47x10^{-1}	5.00x10^{-3}	$<$ 2x10^{-16}
T1	9.67x10^{-3}	1.75x10^{-1}	9.56x10^{-1}	-1.21x10^{-2}	4.36x10^{-3}	5.56x10^{-3}
T10	-2.58x10^{-4}	1.55x10^{-1}	9.99x10^{-1}	8.30x10^{-2}	3.86x10^{-3}	$<$ 2x10^{-16}
T11	-8.50x10^{-2}	1.48x10^{-1}	5.66x10^{-1}	-1.43x10^{-1}	4.52x10^{-3}	$<$ 2x10^{-16}
T12	2.98x10^{-1}	1.68x10^{-1}	7.66x10^{-2}	1.51x10^{-1}	4.14x10^{-3}	$<$ 2x10^{-16}
T13	1.36x10^{-1}	1.56x10^{-1}	3.86x10^{-1}	1.93x10^{-1}	3.91x10^{-3}	$<$ 2x10^{-16}
T14	1.17x10^{-1}	1.58x10^{-1}	4.57x10^{-1}	7.39x10^{-2}	4.14x10^{-3}	$<$ 2x10^{-16}
T15	1.78x10^{-1}	1.90x10^{-1}	3.50x10^{-1}	-1.44x10^{-1}	4.75x10^{-3}	$<$ 2x10^{-16}
T16	3.34x10^{-1}	1.54x10^{-1}	3.04x10^{-2}	3.67x10^{-1}	4.66x10^{-3}	$<$ 2x10^{-16}
T2	-1.47x10^{-1}	1.67x10^{-1}	3.80x10^{-1}	2.19x10^{-1}	4.43x10^{-3}	$<$ 2x10^{-16}
T3	-3.53x10^{-1}	1.60x10^{-1}	2.68x10^{-2}	5.55x10^{-1}	3.84x10^{-3}	$<$ 2x10^{-16}
T4	-1.41x10^{-1}	1.57x10^{-1}	3.69x10^{-1}	-5.23x10^{-2}	5.55x10^{-3}	$<$ 2x10^{-16}
T5	-4.01x10^{-1}	2.04x10^{-1}	4.94x10^{-2}	6.76x10^{-1}	5.02x10^{-3}	$<$ 2x10^{-16}
T6	9.12x10^{-2}	1.49x10^{-1}	5.40x10^{-1}	2.32x10^{-1}	3.88x10^{-3}	$<$ 2x10^{-16}
T7	-2.23x10^{-1}	1.49x10^{-1}	1.34x10^{-1}	1.51x10^{-1}	4.17x10^{-3}	$<$ 2x10^{-16}
T8	7.61x10^{-2}	1.57x10^{-1}	6.27x10^{-1}	5.57x10^{-1}	4.21x10^{-3}	$<$ 2x10^{-16}
T9	2.82x10^{-2}	1.58x10^{-1}	8.58x10^{-1}	-8.14x10^{-3}	4.21x10^{-3}	5.31x10^{-2}
R-squared	1.00x10^{-1}			4.10x10^{-1}		
Log-likelihood	9.63x10^{+3}					
phi	5.44x10^{-1}	8.78x10^{-3}	$<$ 2x10^{-16}			
F-statistic				4.70x10^{+4}		
F-statistic DF				6.70x10^{+1}		

Supplementary Table 5: Regression results for clonal accuracy and SNV CP error. β regressions were used for accuracy and a linear model was used on inverse-normal transformed errors. Two-sided GLM Wald p-values are shown for accuracy and two-sided linear regression p-values are shown for errors. N=4968 and N=4462484, respectively.

	Estimate Accuracy	Std. Error Accuracy	P-value Accuracy	Estimate Error	Std. Error Error	P-value Error
(Intercept)	-6.24×10^{-2}	1.48×10^{-1}	6.72×10^{-1}	-2.21×10^{-1}	3.81×10^{-3}	$< 2\times10^{-16}$
clonal loss	2.88×10^{-1}	7.41×10^{-2}	1.03×10^{-4}	2.43×10^{-1}	1.40×10^{-3}	$< 2\times10^{-16}$
clonal loss*SNV subclonal	-2.60×10^{-1}	1.05×10^{-1}	1.29×10^{-2}	-4.50×10^{-2}	2.80×10^{-3}	$< 2\times10^{-16}$
clonal gain	6.81×10^{-2}	8.90×10^{-2}	4.44×10^{-1}	-5.29×10^{-2}	1.40×10^{-3}	$< 2\times10^{-16}$
clonal gain*SNV subclonal	5.33×10^{-2}	1.21×10^{-1}	6.60×10^{-1}	-9.99×10^{-2}	2.06×10^{-3}	$< 2\times10^{-16}$
subclonal loss	-2.63×10^{-1}	8.06×10^{-2}	1.09×10^{-3}	-1.64×10^{-1}	1.69×10^{-3}	$< 2\times10^{-16}$
subclonal loss*SNV subclonal	3.29×10^{-1}	1.11×10^{-1}	3.06×10^{-3}	1.43×10^{-1}	2.75×10^{-3}	$< 2\times10^{-16}$
subclonal gain	1.77×10^{-1}	8.71×10^{-2}	4.21×10^{-2}	6.24×10^{-3}	2.11×10^{-3}	3.11×10^{-3}
subclonal gain*SNV subclonal	-3.18×10^{-1}	1.20×10^{-1}	7.98×10^{-3}	2.03×10^{-1}	3.63×10^{-3}	$< 2\times10^{-16}$
SNV subclonal	-1.44×10^{-1}	7.28×10^{-2}	4.82×10^{-2}	7.47×10^{-1}	1.16×10^{-3}	$< 2\times10^{-16}$
6052744	5.68×10^{-1}	1.17×10^{-1}	1.21×10^{-6}	-1.50	2.15×10^{-3}	$< 2\times10^{-16}$
6087270	6.59×10^{-1}	1.15×10^{-1}	9.77×10^{-9}	-1.37	2.06×10^{-3}	$< 2\times10^{-16}$
6087309	6.92×10^{-1}	1.20×10^{-1}	6.97×10^{-9}	-4.88×10^{-1}	2.32×10^{-3}	$< 2\times10^{-16}$
6087362	7.38×10^{-1}	1.15×10^{-1}	1.40×10^{-10}	-1.81×10^{-1}	2.05×10^{-3}	$< 2\times10^{-16}$
6181022	4.68×10^{-2}	1.16×10^{-1}	6.86×10^{-1}	-9.63×10^{-1}	2.00×10^{-3}	$< 2\times10^{-16}$
6184474	7.20×10^{-1}	1.14×10^{-1}	2.65×10^{-10}	-2.96×10^{-1}	2.00×10^{-3}	$< 2\times10^{-16}$
6184478	7.24×10^{-1}	1.14×10^{-1}	2.10×10^{-10}	-2.36×10^{-1}	2.00×10^{-3}	$< 2\times10^{-16}$
6184572	6.73×10^{-1}	1.15×10^{-1}	4.24×10^{-9}	-3.03×10^{-1}	2.04×10^{-3}	$< 2\times10^{-16}$
6184761	9.40×10^{-3}	1.20×10^{-1}	9.38×10^{-1}	3.98×10^{-1}	2.19×10^{-3}	$< 2\times10^{-16}$
6185634	7.97×10^{-1}	1.20×10^{-1}	3.02×10^{-11}	5.76×10^{-2}	2.31×10^{-3}	$< 2\times10^{-16}$
6187040	7.49×10^{-1}	1.22×10^{-1}	9.65×10^{-10}	-1.63×10^{-1}	2.36×10^{-3}	$< 2\times10^{-16}$
6187081	5.56×10^{-1}	1.19×10^{-1}	2.77×10^{-6}	-2.16×10^{-1}	2.11×10^{-3}	$< 2\times10^{-16}$
6203106	5.00×10^{-1}	1.29×10^{-1}	1.06×10^{-4}	-4.05×10^{-1}	2.37×10^{-3}	$< 2\times10^{-16}$
6400919	5.86×10^{-1}	1.15×10^{-1}	3.17×10^{-7}	-4.53×10^{-1}	2.00×10^{-3}	$< 2\times10^{-16}$
SOTA1	6.72×10^{-1}	1.15×10^{-1}	4.86×10^{-9}	-7.22×10^{-2}	2.06×10^{-3}	$< 2\times10^{-16}$
SOTA2	7.45×10^{-1}	1.15×10^{-1}	1.06×10^{-10}	-2.36×10^{-1}	2.19×10^{-3}	$< 2\times10^{-16}$
PCAWG-IR	5.55×10^{-1}	1.15×10^{-1}	1.33×10^{-6}	-1.15×10^{-1}	2.00×10^{-3}	$< 2\times10^{-16}$
6181088	7.41×10^{-1}	1.14×10^{-1}	7.48×10^{-11}	6.18×10^{-1}	2.00×10^{-3}	$< 2\times10^{-16}$
6613462	8.28×10^{-2}	1.24×10^{-1}	5.03×10^{-1}	-6.63×10^{-1}	2.11×10^{-3}	$< 2\times10^{-16}$
P10	-1.28×10^{-1}	1.50×10^{-1}	3.94×10^{-1}	-1.82×10^{-1}	3.98×10^{-3}	$< 2\times10^{-16}$
P11	-2.24×10^{-1}	2.02×10^{-1}	2.68×10^{-1}	5.07×10^{-1}	3.89×10^{-3}	$< 2\times10^{-16}$
P12	2.35×10^{-1}	1.88×10^{-1}	2.11×10^{-1}	4.37×10^{-2}	5.36×10^{-3}	3.47×10^{-16}
P13	4.92×10^{-1}	1.55×10^{-1}	1.52×10^{-3}	2.31×10^{-1}	4.14×10^{-3}	$< 2\times10^{-16}$
P14	1.75×10^{-1}	2.10×10^{-1}	4.04×10^{-1}	2.76×10^{-1}	3.84×10^{-3}	$< 2\times10^{-16}$
P15	3.81×10^{-1}	1.68×10^{-1}	2.34×10^{-2}	-6.73×10^{-2}	5.97×10^{-3}	$< 2\times10^{-16}$
P16	2.44×10^{-1}	1.67×10^{-1}	1.43×10^{-1}	1.90×10^{-1}	3.95×10^{-3}	$< 2\times10^{-16}$
P17	3.22×10^{-1}	1.64×10^{-1}	4.98×10^{-2}	1.11×10^{-1}	4.26×10^{-3}	$< 2\times10^{-16}$
P18	9.68×10^{-3}	1.91×10^{-1}	9.59×10^{-1}	5.78×10^{-2}	4.45×10^{-3}	$< 2\times10^{-16}$
P19	5.10×10^{-1}	2.59×10^{-1}	4.90×10^{-2}	2.02×10^{-2}	6.09×10^{-3}	9.16×10^{-4}
P20	5.62×10^{-1}	1.62×10^{-1}	5.36×10^{-4}	-1.26×10^{-1}	6.16×10^{-3}	$< 2\times10^{-16}$
P21	7.89×10^{-2}	1.69×10^{-1}	6.41×10^{-1}	2.03×10^{-1}	3.90×10^{-3}	$< 2\times10^{-16}$
P22	-7.42×10^{-4}	1.55×10^{-1}	9.96×10^{-1}	1.77×10^{-1}	4.55×10^{-3}	$< 2\times10^{-16}$
P23	1.25×10^{-2}	1.56×10^{-1}	9.36×10^{-1}	2.30×10^{-1}	4.00×10^{-3}	$< 2\times10^{-16}$
P24	1.61×10^{-1}	1.73×10^{-1}	3.51×10^{-1}	1.66×10^{-1}	4.61×10^{-3}	$< 2\times10^{-16}$
P25	3.69×10^{-1}	1.57×10^{-1}	1.89×10^{-2}	2.40×10^{-1}	3.83×10^{-3}	$< 2\times10^{-16}$
P3	3.16×10^{-1}	1.84×10^{-1}	8.67×10^{-2}	-7.09×10^{-2}	6.28×10^{-3}	$< 2\times10^{-16}$
P4	3.06×10^{-1}	1.95×10^{-1}	1.16×10^{-1}	-1.96×10^{-1}	6.27×10^{-3}	$< 2\times10^{-16}$
P5	3.98×10^{-1}	1.66×10^{-1}	1.67×10^{-2}	6.49×10^{-2}	4.45×10^{-3}	$< 2\times10^{-16}$
P6	-1.09×10^{-1}	2.20×10^{-1}	6.20×10^{-1}	4.99×10^{-1}	3.88×10^{-3}	$< 2\times10^{-16}$
P8	2.49×10^{-1}	1.48×10^{-1}	9.29×10^{-2}	2.67×10^{-1}	4.06×10^{-3}	$< 2\times10^{-16}$
P9	2.85×10^{-1}	1.68×10^{-1}	8.91×10^{-2}	2.29×10^{-1}	3.88×10^{-3}	$< 2\times10^{-16}$
T0	-7.65×10^{-2}	1.56×10^{-1}	6.24×10^{-1}	-1.47×10^{-1}	5.00×10^{-3}	$< 2\times10^{-16}$
T1	9.67×10^{-3}	1.75×10^{-1}	9.56×10^{-1}	-1.21×10^{-2}	4.36×10^{-3}	5.56×10^{-3}
T10	-2.58×10^{-4}	1.55×10^{-1}	9.99×10^{-1}	8.30×10^{-2}	3.86×10^{-3}	$< 2\times10^{-16}$
T11	-8.50×10^{-2}	1.48×10^{-1}	5.66×10^{-1}	-1.43×10^{-1}	4.52×10^{-3}	$< 2\times10^{-16}$
T12	2.98×10^{-1}	1.68×10^{-1}	7.66×10^{-2}	1.51×10^{-1}	4.14×10^{-3}	$< 2\times10^{-16}$
T13	1.36×10^{-1}	1.56×10^{-1}	3.86×10^{-1}	1.93×10^{-1}	3.91×10^{-3}	$< 2\times10^{-16}$
T14	1.17×10^{-1}	1.58×10^{-1}	4.57×10^{-1}	7.39×10^{-2}	4.14×10^{-3}	$< 2\times10^{-16}$
T15	1.78×10^{-1}	1.90×10^{-1}	3.50×10^{-1}	-1.44×10^{-1}	4.75×10^{-3}	$< 2\times10^{-16}$
T16	3.34×10^{-1}	1.54×10^{-1}	3.04×10^{-2}	3.67×10^{-1}	4.66×10^{-3}	$< 2\times10^{-16}$
T2	-1.47×10^{-1}	1.67×10^{-1}	3.80×10^{-1}	2.19×10^{-1}	4.43×10^{-3}	$< 2\times10^{-16}$
T3	-3.53×10^{-1}	1.60×10^{-1}	2.68×10^{-2}	5.55×10^{-1}	3.84×10^{-3}	$< 2\times10^{-16}$
T4	-1.41×10^{-1}	1.57×10^{-1}	3.69×10^{-1}	-5.23×10^{-2}	5.55×10^{-3}	$< 2\times10^{-16}$
T5	-4.01×10^{-1}	2.04×10^{-1}	4.94×10^{-2}	6.76×10^{-1}	5.02×10^{-3}	$< 2\times10^{-16}$
T6	9.12×10^{-2}	1.49×10^{-1}	5.40×10^{-1}	2.32×10^{-1}	3.88×10^{-3}	$< 2\times10^{-16}$
T7	-2.23×10^{-1}	1.49×10^{-1}	1.34×10^{-1}	1.51×10^{-1}	4.17×10^{-3}	$< 2\times10^{-16}$
T8	7.61×10^{-2}	1.57×10^{-1}	6.27×10^{-1}	5.57×10^{-1}	4.21×10^{-3}	$< 2\times10^{-16}$
T9	2.82×10^{-2}	1.58×10^{-1}	8.58×10^{-1}	-8.14×10^{-3}	4.21×10^{-3}	5.31×10^{-2}
R-squared	1.00×10^{-1}			4.10×10^{-1}		
Log-likelihood	$9.63\times10^{+3}$					
phi	5.44×10^{-1}	8.78×10^{-3}	$< 2\times10^{-16}$			
F-statistic				$4.70\times10^{+4}$		
F-statistic DF				$6.70\times10^{+1}$		

Supplementary Table 6: Regression results assessing the effect of Battenberg CNA accuracy clonal accuracy and SNV CP error in subclonal CNAs. β regressions were used for accuracy and a linear model was used on inverse-normal transformed errors. Two-sided GLM Wald p-values are shown for accuracy and two-sided linear regression p-values are shown for errors. N=5212 and N=4462484, respectively.

	Estimate Accuracy	Std. Error Accuracy	P-value Accuracy	Estimate Error	Std. Error Error	P-value Error
(Intercept)	-1.91×10^{-1}	1.39×10^{-1}	1.69×10^{-1}	6.77×10^{-1}	4.04×10^{-3}	$< 2\times10^{-16}$
Battenberg incorrect	6.02×10^{-2}	7.78×10^{-2}	4.39×10^{-1}	1.12×10^{-1}	2.73×10^{-3}	$< 2\times10^{-16}$
clonal loss	2.75×10^{-2}	7.61×10^{-2}	7.17×10^{-1}	2.12×10^{-1}	2.65×10^{-3}	$< 2\times10^{-16}$
clonal loss*SNV clonal	2.55×10^{-1}	1.05×10^{-1}	1.49×10^{-2}	4.27×10^{-2}	2.97×10^{-3}	$< 2\times10^{-16}$
clonal gain	1.24×10^{-1}	9.00×10^{-2}	1.69×10^{-1}	-1.52×10^{-1}	2.09×10^{-3}	$< 2\times10^{-16}$
clonal gain*SNV clonal	-5.26×10^{-2}	1.21×10^{-1}	6.65×10^{-1}	1.01×10^{-1}	2.18×10^{-3}	$< 2\times10^{-16}$
subclonal loss	2.99×10^{-2}	7.87×10^{-2}	7.05×10^{-1}	-2.74×10^{-2}	2.50×10^{-3}	$< 2\times10^{-16}$
subclonal loss*SNV clonal	-2.59×10^{-1}	1.08×10^{-1}	1.58×10^{-2}	-1.53×10^{-1}	2.92×10^{-3}	$< 2\times10^{-16}$
subclonal gain	-2.25×10^{-1}	8.85×10^{-2}	1.12×10^{-2}	1.85×10^{-1}	3.58×10^{-3}	$< 2\times10^{-16}$
subclonal gain*SNV clonal	4.56×10^{-1}	1.15×10^{-1}	7.75×10^{-5}	-2.33×10^{-1}	3.85×10^{-3}	$< 2\times10^{-16}$
SNV clonal	1.40×10^{-1}	7.30×10^{-2}	5.43×10^{-2}	-8.00×10^{-1}	1.23×10^{-3}	$< 2\times10^{-16}$
6052744	5.38×10^{-1}	1.14×10^{-1}	2.53×10^{-6}	-1.58	2.28×10^{-3}	$< 2\times10^{-16}$
6087270	6.33×10^{-1}	1.12×10^{-1}	1.71×10^{-8}	-1.43	2.19×10^{-3}	$< 2\times10^{-16}$
6087309	6.62×10^{-1}	1.17×10^{-1}	1.53×10^{-8}	-5.29×10^{-1}	2.46×10^{-3}	$< 2\times10^{-16}$
6087362	7.01×10^{-1}	1.12×10^{-1}	4.50×10^{-10}	-2.02×10^{-1}	2.17×10^{-3}	$< 2\times10^{-16}$
6181022	4.25×10^{-2}	1.13×10^{-1}	7.07×10^{-1}	-1.01	2.12×10^{-3}	$< 2\times10^{-16}$
6184474	6.81×10^{-1}	1.11×10^{-1}	9.60×10^{-10}	-3.30×10^{-1}	2.12×10^{-3}	$< 2\times10^{-16}$
6184478	6.86×10^{-1}	1.11×10^{-1}	7.28×10^{-10}	-2.67×10^{-1}	2.12×10^{-3}	$< 2\times10^{-16}$
6184572	6.33×10^{-1}	1.12×10^{-1}	1.56×10^{-8}	-3.36×10^{-1}	2.16×10^{-3}	$< 2\times10^{-16}$
6184761	-2.57×10^{-4}	1.17×10^{-1}	9.98×10^{-1}	4.32×10^{-1}	2.32×10^{-3}	$< 2\times10^{-16}$
6185634	7.68×10^{-1}	1.18×10^{-1}	8.41×10^{-11}	4.85×10^{-2}	2.44×10^{-3}	$< 2\times10^{-16}$
6187040	7.14×10^{-1}	1.20×10^{-1}	2.81×10^{-9}	-1.76×10^{-1}	2.50×10^{-3}	$< 2\times10^{-16}$
6187081	5.25×10^{-1}	1.16×10^{-1}	5.74×10^{-6}	-2.41×10^{-1}	2.23×10^{-3}	$< 2\times10^{-16}$
6203106	4.66×10^{-1}	1.27×10^{-1}	2.42×10^{-4}	-4.41×10^{-1}	2.52×10^{-3}	$< 2\times10^{-16}$
6400919	5.49×10^{-1}	1.12×10^{-1}	9.34×10^{-7}	-4.94×10^{-1}	2.12×10^{-3}	$< 2\times10^{-16}$
SOTA1	6.35×10^{-1}	1.12×10^{-1}	1.50×10^{-8}	-8.84×10^{-2}	2.19×10^{-3}	$< 2\times10^{-16}$
SOTA2	7.06×10^{-1}	1.13×10^{-1}	3.75×10^{-10}	-2.63×10^{-1}	2.32×10^{-3}	$< 2\times10^{-16}$
PCAWG-IR	5.28×10^{-1}	1.12×10^{-1}	2.50×10^{-6}	-1.32×10^{-1}	2.12×10^{-3}	$< 2\times10^{-16}$
6181088	7.04×10^{-1}	1.11×10^{-1}	2.47×10^{-10}	6.53×10^{-1}	2.12×10^{-3}	$< 2\times10^{-16}$
6613462	8.12×10^{-2}	1.21×10^{-1}	5.01×10^{-1}	-7.08×10^{-1}	2.23×10^{-3}	$< 2\times10^{-16}$
P10	-1.28×10^{-1}	1.42×10^{-1}	3.67×10^{-1}	-1.93×10^{-1}	4.22×10^{-3}	$< 2\times10^{-16}$
P11	-2.14×10^{-1}	1.96×10^{-1}	2.75×10^{-1}	5.25×10^{-1}	4.13×10^{-3}	$< 2\times10^{-16}$
P12	2.39×10^{-1}	1.82×10^{-1}	1.91×10^{-1}	5.19×10^{-2}	5.68×10^{-3}	$< 2\times10^{-16}$
P13	5.05×10^{-1}	1.48×10^{-1}	6.48×10^{-4}	2.42×10^{-1}	4.40×10^{-3}	$< 2\times10^{-16}$
P14	1.78×10^{-1}	2.05×10^{-1}	3.85×10^{-1}	2.94×10^{-1}	4.07×10^{-3}	$< 2\times10^{-16}$
P15	3.88×10^{-1}	1.61×10^{-1}	1.61×10^{-2}	-6.72×10^{-2}	6.33×10^{-3}	$< 2\times10^{-16}$
P16	2.50×10^{-1}	1.61×10^{-1}	1.20×10^{-1}	2.00×10^{-1}	4.19×10^{-3}	$< 2\times10^{-16}$
P17	3.27×10^{-1}	1.57×10^{-1}	3.75×10^{-2}	1.16×10^{-1}	4.52×10^{-3}	$< 2\times10^{-16}$
P18	1.59×10^{-2}	1.85×10^{-1}	9.31×10^{-1}	5.02×10^{-2}	4.72×10^{-3}	$< 2\times10^{-16}$
P19	5.11×10^{-1}	2.55×10^{-1}	4.50×10^{-2}	1.94×10^{-2}	6.46×10^{-3}	2.66×10^{-3}
P20	5.66×10^{-1}	1.55×10^{-1}	2.54×10^{-4}	-1.27×10^{-1}	6.53×10^{-3}	$< 2\times10^{-16}$
P21	8.29×10^{-2}	1.63×10^{-1}	6.10×10^{-1}	2.09×10^{-1}	4.13×10^{-3}	$< 2\times10^{-16}$
P22	-2.03×10^{-2}	1.49×10^{-1}	8.92×10^{-1}	1.68×10^{-1}	4.87×10^{-3}	$< 2\times10^{-16}$
P23	1.86×10^{-2}	1.48×10^{-1}	9.00×10^{-1}	2.34×10^{-1}	4.24×10^{-3}	$< 2\times10^{-16}$
P24	1.67×10^{-1}	1.66×10^{-1}	3.14×10^{-1}	1.67×10^{-1}	4.89×10^{-3}	$< 2\times10^{-16}$
P25	3.74×10^{-1}	1.49×10^{-1}	1.24×10^{-2}	2.52×10^{-1}	4.07×10^{-3}	$< 2\times10^{-16}$
P3	3.19×10^{-1}	1.78×10^{-1}	7.42×10^{-2}	-8.90×10^{-2}	6.65×10^{-3}	$< 2\times10^{-16}$
P4	3.14×10^{-1}	1.90×10^{-1}	9.73×10^{-2}	-2.14×10^{-1}	6.64×10^{-3}	$< 2\times10^{-16}$
P5	3.99×10^{-1}	1.59×10^{-1}	1.23×10^{-2}	6.34×10^{-2}	4.72×10^{-3}	$< 2\times10^{-16}$
P6	-1.31×10^{-1}	2.16×10^{-1}	5.45×10^{-1}	5.13×10^{-1}	4.12×10^{-3}	$< 2\times10^{-16}$
P8	2.57×10^{-1}	1.40×10^{-1}	6.73×10^{-2}	2.83×10^{-1}	4.30×10^{-3}	$< 2\times10^{-16}$
P9	2.87×10^{-1}	1.61×10^{-1}	7.44×10^{-2}	2.43×10^{-1}	4.11×10^{-3}	$< 2\times10^{-16}$
T0	-6.87×10^{-2}	1.48×10^{-1}	6.43×10^{-1}	-1.66×10^{-1}	5.30×10^{-3}	$< 2\times10^{-16}$
T1	1.19×10^{-2}	1.56×10^{-1}	9.39×10^{-1}	-3.33×10^{-2}	4.64×10^{-3}	7.40×10^{-13}
T10	-1.79×10^{-2}	1.41×10^{-1}	8.99×10^{-1}	8.55×10^{-2}	4.10×10^{-3}	$< 2\times10^{-16}$
T11	-8.86×10^{-2}	1.40×10^{-1}	5.26×10^{-1}	-1.61×10^{-1}	4.79×10^{-3}	$< 2\times10^{-16}$
T12	3.01×10^{-1}	1.61×10^{-1}	6.13×10^{-2}	1.55×10^{-1}	4.39×10^{-3}	$< 2\times10^{-16}$
T13	1.44×10^{-1}	1.49×10^{-1}	3.33×10^{-1}	2.03×10^{-1}	4.15×10^{-3}	$< 2\times10^{-16}$
T14	1.26×10^{-1}	1.51×10^{-1}	4.05×10^{-1}	7.73×10^{-2}	4.39×10^{-3}	$< 2\times10^{-16}$
T15	1.84×10^{-1}	1.84×10^{-1}	3.17×10^{-1}	-1.61×10^{-1}	5.04×10^{-3}	$< 2\times10^{-16}$
T16	3.29×10^{-1}	1.46×10^{-1}	2.45×10^{-2}	3.79×10^{-1}	4.94×10^{-3}	$< 2\times10^{-16}$
T2	-1.36×10^{-1}	1.60×10^{-1}	3.95×10^{-1}	2.26×10^{-1}	4.69×10^{-3}	$< 2\times10^{-16}$
T3	-3.46×10^{-1}	1.38×10^{-1}	1.20×10^{-2}	5.74×10^{-1}	4.07×10^{-3}	$< 2\times10^{-16}$
T4	-1.28×10^{-1}	1.50×10^{-1}	3.95×10^{-1}	-5.11×10^{-2}	5.89×10^{-3}	$< 2\times10^{-16}$
T5	-4.02×10^{-1}	1.74×10^{-1}	2.07×10^{-2}	6.80×10^{-1}	5.41×10^{-3}	$< 2\times10^{-16}$
T6	9.70×10^{-2}	1.41×10^{-1}	4.92×10^{-1}	2.40×10^{-1}	4.11×10^{-3}	$< 2\times10^{-16}$
T7	-2.07×10^{-1}	1.35×10^{-1}	1.24×10^{-1}	1.59×10^{-1}	4.42×10^{-3}	$< 2\times10^{-16}$
T8	8.55×10^{-2}	1.50×10^{-1}	5.68×10^{-1}	5.83×10^{-1}	4.46×10^{-3}	$< 2\times10^{-16}$
T9	3.88×10^{-2}	1.50×10^{-1}	7.96×10^{-1}	-1.28×10^{-2}	4.46×10^{-3}	4.05×10^{-3}
R-squared	1.00×10^{-1}			4.10×10^{-1}		
Log-likelihood	$1.03\times10^{+4}$					
phi	5.30×10^{-1}	8.31×10^{-3}	$< 2\times10^{-16}$			
F-statistic				$4.59\times10^{+4}$		
F-statistic DF				$6.80\times10^{+1}$		
