# Supplementary material for: Crowd-sourced benchmarking of single-sample tumor subclonal reconstruction
Source: Nat Biotechnol. 2024 Jun 11;43(4):581–92. doi: 10.1038/s41587-024-02250-y (PMC11994449; doi:10.1038/s41587-024-02250-y)
Supplement: Supplementary file 2 — Reporting Summary [file 41587_2024_2250_MOESM2_ESM.pdf]

Corresponding author(s): Paul C. Boutros
Last updated by author(s): Apr 6, 2024

Reporting Summary

Nature Portfolio wishes to improve the reproducibility of the work that we publish. This form provides structure for consistency and transparency in reporting. For further information on Nature Portfolio policies, see our Editorial Policies and the Editorial Policy Checklist.

Statistics

For all statistical analyses, confirm that the following items are present in the figure legend, table legend, main text, or Methods section.

n/a	Confirmed	
☐	☒	The exact sample size (*n*) for each experimental group/condition, given as a discrete number and unit of measurement
☐	☒	A statement on whether measurements were taken from distinct samples or whether the same sample was measured repeatedly
☐	☒	The statistical test(s) used AND whether they are one- or two-sided<br>*Only common tests should be described solely by name; describe more complex techniques in the Methods section.*
☐	☒	A description of all covariates tested
☐	☒	A description of any assumptions or corrections, such as tests of normality and adjustment for multiple comparisons
☐	☒	A full description of the statistical parameters including central tendency (e.g. means) or other basic estimates (e.g. regression coefficient) AND variation (e.g. standard deviation) or associated estimates of uncertainty (e.g. confidence intervals)
☐	☒	For null hypothesis testing, the test statistic (e.g. *F*, *t*, *r*) with confidence intervals, effect sizes, degrees of freedom and *P* value noted<br>*Give P values as exact values whenever suitable.*
☒	☐	For Bayesian analysis, information on the choice of priors and Markov chain Monte Carlo settings
☒	☐	For hierarchical and complex designs, identification of the appropriate level for tests and full reporting of outcomes
☐	☒	Estimates of effect sizes (e.g. Cohen's *d*, Pearson's *r*), indicating how they were calculated

Our web collection on statistics for biologists contains articles on many of the points above.

Software and code

Policy information about availability of computer code

Data collection	No software was used to download the data used in this publication.
Data analysis	All our custom code were deposited in public repositories. BAMSurgeon is available at: https://github.com/adamewing/bamsurgeon. The framework for subclonal mutation simulation is available at: http://search.cpan.org/~boutroslb/NGS-Tools-BAMSurgeonv1.0.0/. The PhaseTools BAM phasing toolkit is available at https://github.com/mateidavid/phase-tools. Scripts providing the complete scoring harness are available at: ://github.com/uclahs-cds/tool-SMCHet-scoring. R, lattice, latticeExtra, gridExtra, gtable BPG and betareg are available through https://cran.r-project.org. Docker containers of submissions were deposited at https://www.synapse.org/#!Synapse:syn2813581/docker/ and Galaxy workflows at https://github.com/smc-het-challenge/ . MOBSTER is available at https://github.com/caravagnalab/mobster . DPClust is available at https://github.com/Wedge-lab/dpclust3p (commit a505664). Battenberg (v2.2.10) is available at https://github.com/Wedge-lab/battenberg . PhyloWGS is available at https://github.com/morrislab/phylowgs (commit 3e21cec). Mutect is available athrough GATK at https://gatk.broadinstitute.org .

For manuscripts utilizing custom algorithms or software that are central to the research but not yet described in published literature, software must be made available to editors and reviewers. We strongly encourage code deposition in a community repository (e.g. GitHub). See the Nature Portfolio guidelines for submitting code & software for further information.

Data

Policy information about availability of data

All manuscripts must include a data availability statement. This statement should provide the following information, where applicable:

- Accession codes, unique identifiers, or web links for publicly available datasets
- A description of any restrictions on data availability
- For clinical datasets or third party data, please ensure that the statement adheres to our policy

BAM files are available in EGA at EGAS00001002092. SNV, SV, CNA, and Indel calls and corresponding truth files are available at https://www.synapse.org/#!Synapse:syn2813581/files/. The normal BAM with spiked in mutations is available at https://www.ebi.ac.uk/ena/browser/view/PRJEB52520. Human genome assembly hs37d5 was used as the reference. Scores are available for download at https://mtarabichi.shinyapps.io/smchet_results/. Figures 1-6 show data analyses based on scores and simulated BAMs.

Human research participants

Policy information about studies involving human research participants and Sex and Gender in Research.

Reporting on sex and gender	No human research participants are used, all data is synthetic.
Population characteristics	No human research participants are used, all data is synthetic.
Recruitment	No human research participants are used, all data is synthetic.
Ethics oversight	NA

Note that full information on the approval of the study protocol must also be provided in the manuscript.

Field-specific reporting

Please select the one below that is the best fit for your research. If you are not sure, read the appropriate sections before making your selection.

☒ Life sciences ☐ Behavioural & social sciences ☐ Ecological, evolutionary & environmental sciences

For a reference copy of the document with all sections, see nature.com/documents/nr-reporting-summary-flat.pdf

Life sciences study design

All studies must disclose on these points even when the disclosure is negative.

Sample size	We based our analysis on 51 previously published simulated tumors that covered a wide range of tumor types, mutation burden, read-depths and tree topologies that would enable us to investigate correlation between algorithm performance and tumor features. We also used 5 simulated tumors that had been downsampled to 5 depths which had been shown to effectively demonstrate the effect of read-depth on performance (Salcedo et al., Nature Biotechnology, 2020). We obtained 31 dockerized workflows through a crowd-based DREAM Challenge. The challenge was run in accordance to DREAM protocol and in collaboration with the PCAWG Heterogeneity working group to encourage participation from a wide range of teams. We supplemented these with 3 informative baselines (one-cluster, random, and informed random algorithms) and two established algorithms for each SubChallenge.
Data exclusions	We excluded submissions from a given team that were strongly correlated (r > 0.75) to ensure the independence of observations in our analyses. Similarly, for most analyses we excluded the 10 'corner-case' tumors that were all based on a single tree topology, except where otherwise stated. We also excluded two tumors with exceptional mutation burden (> 100K SNVs) where only five algorithms successfully produced outputs to ensure they did not bias our results as outliers.
Replication	Due to the nature of simulation-based studies, for which the truth is known, we implemented quality checks and systematic comparisons to the truth to assess the reproducibility and validity of the results. All statistical tests were run across multiple independent tumours and/or algorithms as appropriate. We compared scores from original submissions to updated algorithms submitted by developers for a subset of algorithms and observed scores were consistent (Extended Data Figure 2). To assess the generalizability of our rankings, we used bootstrap resampling to draw 1000 subsets of tumours and re-calculated our rankings. We report bootstrap confidence intervals for the median score and ranking of each algorithm (Extended Data Figure 4). We also compared scores and predictions from a subset of algorithms on four of the titration series tumors before and after adding neutral mutations evolving through a branching process. We found they were largely consistent and lacked directional biases (Extended Data Figure 8 and 9). When testing for the effects of algorithmic neutral mutation filtration, we compared the results from five algorithms on the four titration series tumours (Supplementary Figure 5).
Randomization	Our study did not explicitly derive experimental groups but rather described the performance and error profiles of all algorithms across all tumors. Tumor designs were based on real, published tumors and their features were determined prior to data collection and analysis. Algorithm features were described by developers at submission time.

Blinding	Our study did not explicitly derive experimental groups but rather described the performance and error profiles of all algorithms across all tumors. Tumor designs were based on real, published tumors and their features were determined prior to data collection and analysis. Algorithm features were described by developers at submission time.

Reporting for specific materials, systems and methods

We require information from authors about some types of materials, experimental systems and methods used in many studies. Here, indicate whether each material, system or method listed is relevant to your study. If you are not sure if a list item applies to your research, read the appropriate section before selecting a response.

Materials & experimental systems

n/a	Involved in the study	
☒	☐	Antibodies
☒	☐	Eukaryotic cell lines
☒	☐	Palaeontology and archaeology
☒	☐	Animals and other organisms
☒	☐	Clinical data
☒	☐	Dual use research of concern

Methods

n/a	Involved in the study	
☒	☐	ChIP-seq
☒	☐	Flow cytometry
☒	☐	MRI-based neuroimaging
